# High permeability sub-nanometre sieve composite MoS_2_ membranes

**DOI:** 10.1038/s41467-020-16577-y

**Published:** 2020-06-02

**Authors:** Bedanga Sapkota, Wentao Liang, Armin VahidMohammadi, Rohit Karnik, Aleksandr Noy, Meni Wanunu

**Affiliations:** 1 Department of Physics, Northeastern University, Boston, MA 02115 USA; 2 Kostas Advanced Nanocharacterization Facility (KANCF), Northeastern University, Burlington, MA 01803 USA; 3 Department of Materials Engineering, Auburn University, Auburn, AL 36849 USA; 4 Department of Mechanical Engineering, Massachusetts Institute of Technology, 77 Massachusetts Avenue, Cambridge, MA 02139 USA; 5 Physical and Life Sciences Directorate, Lawrence Livermore National Laboratory, 7000 East Avenue, Livermore, CA 94550 USA; 6 School of Natural Sciences, University of California Merced, Merced, CA 95343 USA

**Keywords:** Two-dimensional materials, Nanopores

## Abstract

Two-dimensional membranes have gained enormous interest due to their potential to deliver precision filtration of species with performance that can challenge current desalination membrane platforms. Molybdenum disulfide (MoS_2_) laminar membranes have recently demonstrated superior stability in aqueous environment to their extensively-studied analogs graphene-based membranes; however, challenges such as low ion rejection for high salinity water, low water flux, and low stability over time delay their potential adoption as a viable technology. Here, we report composite laminate multilayer MoS_2_ membranes with stacked heterodimensional one- to two-layer-thick porous nanosheets and nanodisks. These membranes have a multimodal porous network structure with tunable surface charge, pore size, and interlayer spacing. In forward osmosis, our membranes reject more than 99% of salts at high salinities and, in reverse osmosis, small-molecule organic dyes and salts are efficiently filtered. Finally, our membranes stably operate for over a month, implying their potential for use in commercial water purification applications.

## Abstract (web summary)

Molybdenum disulfide membranes are attractive for filtration of nanoscale species but should be optimized for application. Here, the authors report composite membranes with tunable surface charge, pore size and interlayer spacing, achieving efficient filtration of small-molecule dyes and osmosis.

## Introduction

The increasing global need for potable water is a prime global challenge that faces sustainable life on our planet. While water is available in ample quantities on earth, the vast majority (>98%) is in undrinkable form (e.g., seawater, brackish water, or sewage water). Viable and sustainable solutions to this problem demand new materials and processes that can efficiently purify water from contaminated sources, which includes removal of debris, biological matter, organic and inorganic impurities, and various salts. Among these, the most challenging impurities are salt and small neutral organic molecules, because their hydrodynamic size is most comparable to water molecules, complicating size-based separations. To address these issues, low-cost membranes are needed that selectively reject ions and neutral species while still allowing rapid water transport^1,2^, so that water purification becomes energy efficient.

Recently, advanced nanoscale materials have been at the forefront of new water purification technologies. For example, ultrathin, carbon-based two-dimensional (2D) materials such as graphene and graphene oxide (GO) are excellent membrane candidates due to their mechanical/thermal/chemical stability, controllable porosity, and controlled chemical functionality^3–12^. Owing to its low cost and manufacturability, extensive studies on GO membranes have been presented in the literature^13–16^. GO membranes are typically multi-layered, where transport occurs in between stacked GO flake laminates as well as through any pores or defects in or between the flakes. The interlayer spacing, which defines the performance of these membranes, is controlled by crosslinking^13^, casting in epoxy^4^, electrically^17^, and pre-use membrane immersion in various salts for several weeks^5^. While promising, several limitations for these membranes are noted: (1) membrane swelling in water, which lowers ion selectivity due to increased interlayer spacing^4,18^; (2) low water transport due to friction-type interaction between water and functional groups on the graphene surface, which cover 40–60% of the GO surface^19^ and results in only a partial utilization of the 2D channel geometry; and (3) limited lifetime of a few hours before loss of efficacy and/or mechanical failure due to swelling or dissolution^20–22^.

In addition to carbon-based materials, other 2D materials such as molybdenum disulfide (MoS_2_) have recently been explored for water desalination, revealing some distinct features such as zero swelling in water^21,23^ due to a favorable balance between attractive van der Waals between neighboring nanosheets (NSs) and repulsive hydration forces. Simulations also suggest that, due to their crystalline porous structure, these materials maintain robustness while allowing ultrafast water permeance and high ion selectivities^24,25^. A recent comparative simulation reported that single-layer porous MoS_2_ membrane can lead to 70% higher water flux than a porous graphene membrane^26^. Furthermore, nanopores in MoS_2_ are intrinsically charged due to electron redistribution between Mo and S atoms^27^, which can enhance their ion selectivity via repulsive membrane–ion interactions. Recently, MoS_2_ nanopores were used as osmotic power generators, which surpass boron nitride nanotubes^28^. Researchers also demonstrated superior water-transport kinetics for MoS_2_ nanopores over graphene^29^, suggesting a huge potential for nanoporous MoS_2_ for water desalination. Despite strong merits predicted by a number of theoretical studies^26,27,29^, experimental examination of nanoporous MoS_2_ is yet to elucidate and validate its effectiveness in a broader context of membrane technology. One study has reported dye functionalization of MoS_2_ membrane^30^, which requires over a month-long tedious preparation (sandwiching membrane in between dye and water over 21 days and membrane cleaning over 2 weeks). Unfortunately, such process had difficulties controlling interlayer spacing, interlayer configuration, and desalination performance of the resulting membrane was limited to only a few hours.

We report here a straightforward and scalable cavitation-based method to create nanoporous MoS_2_ NSs, resulting in a mixture of one-to-two-layer-thick porous NSs and nanodisks (NDs). Control over the mean nanopore size is achieved by adjusting the processing times, and the surface charge of the porous NS/ND mixture (hereon referred to as NSND) can be tuned in <20 min using custom-designed cationic and anionic polypeptide adsorbent molecules. Laminate membranes (LMs) formed by stacking these nanomaterials on a porous alumina support are highly stable and further demonstrate high ion selectivities and water transport rates in both forward osmosis (FO) and reverse osmosis (RO) modes, as compared to the state-of-the art commercial thin-film composite (TFC) polyamide (PA) membranes. It is noted that, by improving ionic rejection and water transport rate while maintaining a stable performance, the capital and operational cost of RO operation can be lowered^31^. Our approach of producing membranes from controlled size NS/ND/peptide dispersions leads to rationally engineered porosity, where pore size, interlayer spacing, and surface charge can all be tuned. The resulting membranes exhibit high selectivity to water and high water permeance values, due to the presence of pores within NSs, intrasheet spacings, and interspersed NDs, the latter acting to increase the number of sub-nanometer channels (void agents) between larger NSs. Most importantly, combining the advantages of two competing approaches, i.e., ultrathin porous single layer membrane and layer-stacked membrane, scaling up problem could be addressed to realize the practical applications of 2D material-based membranes^32^.

## Results

### Producing porous MoS_2_ NSNDs

**Fig. 1:**
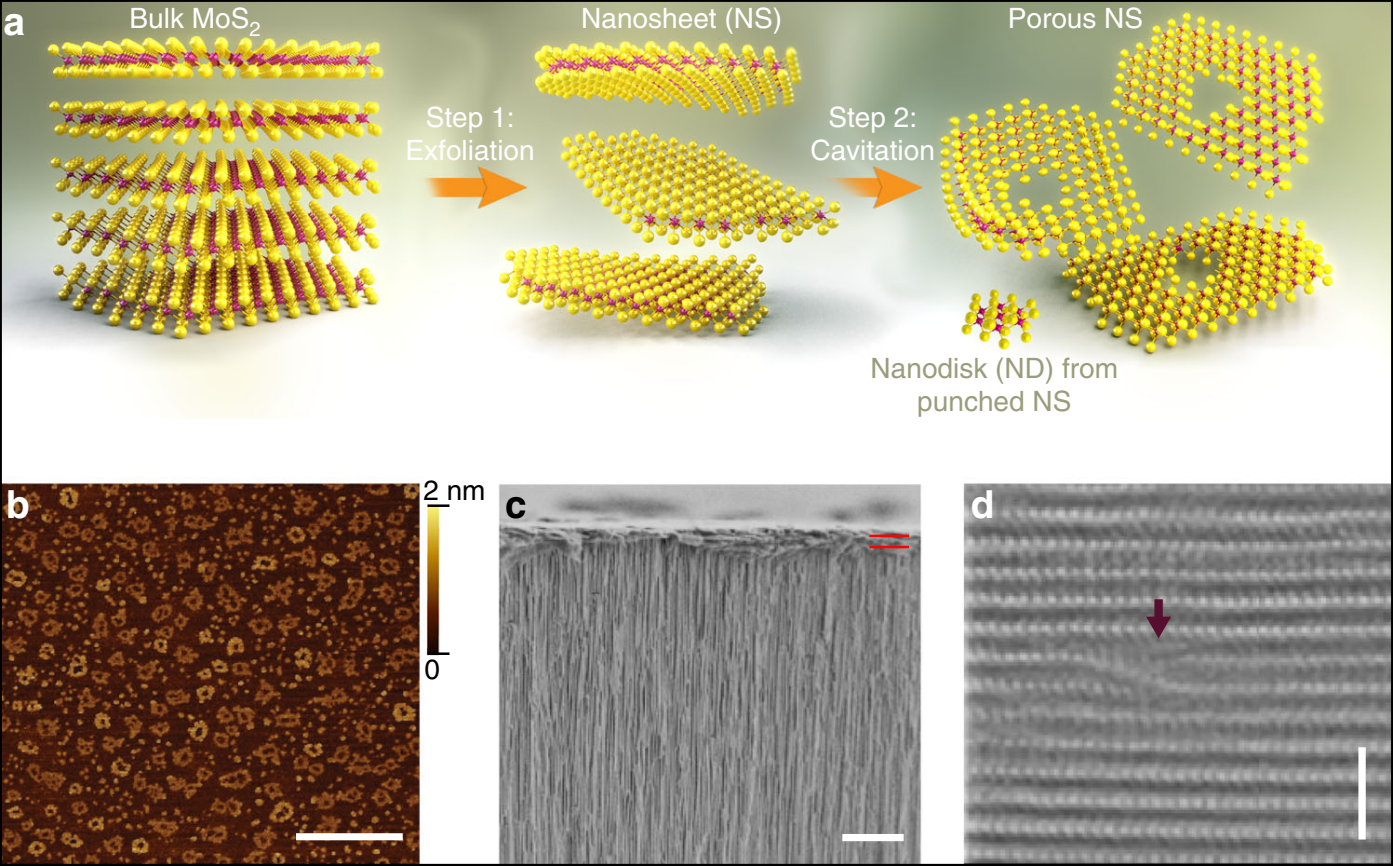
Porous MoS_2_ nanosheets. **a** Schematic representation of a two-step process for the preparation of porous MoS_2_ nanosheets (NSs) and nanodisks (NDs). **b** Topographic atomic force micrographs of as-prepared porous NSs dispersed on freshly cleaved mica (scale bar = 500 nm). **c** Cross-sectional SEM image of a laminate NS/ND membrane supported onto an Anodisc alumina filter, where the approximate 1-µm thickness is highlighted by the two burgundy lines (scale bar = 5 µm). **d** A representative high-resolution HAADF STEM image of a laminate cross-section, showing an interlayer spacing of 6.2 Å and stacking faults induced by nanosheet porosity and intersheet/ND stacking defects. A defect that results in interlayer voids, as shown by the burgundy arrow (scale bar = 2 nm).

Porous MoS_2_ NSNDs were prepared from natural MoS_2_ powder (low cost and earth-abundant material) using a two-step method (Fig. 1a): The first step involves ultrasound-assisted exfoliation and milling of bulk MoS_2_ into thinner and smaller particles at a power of 20 W; this process involves breaking covalent bonds to make smaller sheets, as well as breaking noncovalent interactions between sheets of MoS_2_ that are held together by weak van der Waals forces. The second step involves probe sonication of the NSs, in which the more intense ultrasonic power (500 W) is observed to create nanoholes in the thinner NSs. The high-intensity ultrasound is expected to lead to cavitation, with enhanced heterogeneous nucleation due to the presence of suspended MoS_2_ NSs^33^. Cavitation is a complex phenomenon, but it can lead to extreme conditions including high-shear liquid jets with high velocities^34,35^. These jets and associated shock waves may introduce holes in the MoS_2_ NSs, which may be aided by rapid acceleration and collision of the MoS_2_ particles^34^. Interestingly, we find that the creation of nanoholes is always accompanied by the creation of NDs of similar size to the nanoholes (Supplementary Figs. 1–3), suggesting that the second sonication step “punches out” holes in the NSs from which the NDs are formed^36^. We find that both the mean NS diameters and the mean diameters of the as-created nanoholes in the exfoliated NSs can be controlled by adjusting the sonication duration (Supplementary Table 1). In Fig. 1b, we show an atomic force microscopic (AFM) image of the porous MoS_2_ NSs that were prepared from natural bulk MoS_2_ powder (<2 µm) by a 4-h bath sonication step, followed by a 2-h probe sonication step (see details in the Supplementary Section 1). For this sample, the average NS diameter was 163 ± 20 nm, and the average hole size was 32 ± 8 nm. The dark features in the middle of the NSs are pores formed from knockout of material from within the NSs, whereas the ejected ND products are seen scattered throughout in the image. The height profiles of the NSs follows a binary distribution predominantly of two heights, 0.7 nm and 1.4 nm (Supplementary Fig. 1), corresponding to exactly 1- and 2-layer-thick porous NSs.

### Fabrication of porous MoS_2_ NSND membranes

**Fig. 2:**
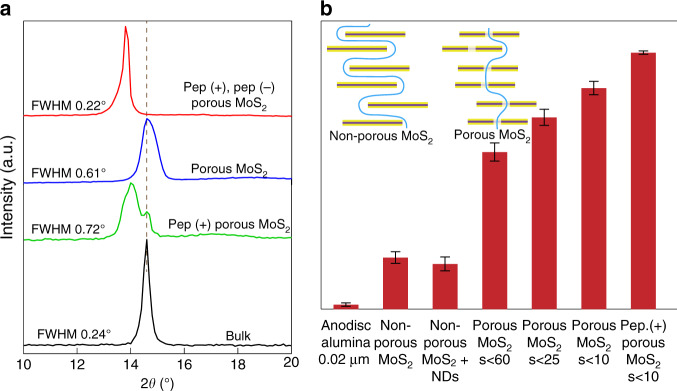
Effect of interlayer arrangement, pore size, nanodisk spacing, and peptide presence on salt rejection. **a** X-ray diffraction for bulk, porous MoS_2_, positive peptide-decorated (pep.+) porous MoS_2_, and a peptide-decorated (pep.+, pep.−) porous MoS_2_ membranes showing the signature of (002) peak position. **b** Comparison of aqueous NaCl (0.5 M) rejection by a bare Anodisc alumina filter, a non-porous MoS_2_ NSL, a non-porous MoS_2_ NSL with NDs, a porous MoS_2_ NSL of different pore diameters, and a peptide-decorated (pep.+, pep.−) porous MoS_2_ NSL. Rejection was measured after a 1-day sucrose-driven permeability experiment (*n* ≥ 3 for all measurements). Inset scheme highlights proposed trajectory of the path of least resistance of water permeation in a porous MoS_2_ NSL in contrast to highly tortuous path in a non-porous MoS_2_ NSL.

We prepared NSND-LMs by vacuum filtration of suspensions with equal nanomaterial volumes and concentrations, as reported previously^37^. This procedure provided similar membrane thicknesses (~1 µm) for our preparations, as confirmed using cross-sectional scanning electron microscopic (SEM) measurements (Fig. 1c). The vertical lines in the image show the supported alumina filter, whereas the NSND-LM is horizontally oriented (parallel red lines indicate ~1-µm laminate film thickness). A closer look at the structure of the LM is seen in Fig. 1d, where high-resolution high-angle annular dark-field scanning transmission electron microscopic (HAADF STEM) image shows stacking faults induced by the heterodimensional structure of the stacked sheets/disks in the LM. In this image, the average spacing between Mo atoms is 6.2 Å, in accordance with X-ray diffraction (XRD; Fig. 2a) and literature values^21,30,38^. We expect that all of these structural features, namely, creation of channels by the NDs and creation of through-pathways by the porous structure of the NSs, as well as the stacking defects within the laminate, play significant roles on water transport kinetics through the LM.

### Tuning surface charge and interlayer spacing in membrane

To understand the effect of surface charge and interaction between NSs, LMs were prepared in the presence of both negatively charged (sequence, EFEFEFEF) and positively charged (sequence, KFKFKFKF) peptides, referred to as pep. (−) and pep. (+) (see Methods section for details about the peptides and preparation steps). The peptides are designed to self-assemble on monolayer MoS_2_ (Supplementary Fig. 7), which is also expected to influence interactions between NSs and LM assembly. The peptides serve two key functions: first, they help with dispersing the NSNDs in water (Supplementary Fig. 6) and therefore facilitate membrane preparation, and second, they modulate the surface charge of the NSNDs by virtue of their charged amino acid residues (Supplementary Fig. 9). To elucidate the effect of peptide on interlayer spacing and interlayer arrangement, we performed XRD of the LMs (Fig. 2a). We observe a slight increase in interlayer spacing from 0.61 to 0.67 nm (~1° shift), in addition to a narrower XRD peak of 0.22° for the membrane with peptide functionalization (full-width at half-maximum), than for porous MoS_2_ (0.61°) and for positively charged MoS_2_ (0.72°), i.e., pep (+) porous MoS_2_. The narrower and sharp XRD peak suggests a more uniform and highly ordered stacking of the oppositely charged NSs throughout the laminate. For the other cases shown in the XRD plot, the misorientation of the flakes with respect to each other is presumably due to stacking defect, which might have caused the broadening of (002) peak of basal planes of MoS_2_. To study the interlayer spacing of wet membranes, we performed XRD of porous MoS_2_ and a peptide-decorated (pep.+, pep.−) porous MoS_2_ re-wetted membranes (first baked at 50 °C and re-wetted, see Supplementary Section 1 for details) and observed increasing in interlayer spacing by 0.17 nm upon peptide incorporation (Supplementary Fig. 8). To understand the role of pores in the NSs and the NDs in determining transport properties, we prepared LMs consisting of only NSs (without the pore-creating second step), NSs without pores but with NDs, and NSNDs under different degrees of probe sonication (Supplementary Table 1), which affords systematic control of the membrane properties.

### Nanofiltration

We sandwiched our ~1-µm-thick porous NSND-LMs in between feed and permeate compartments and performed salt-rejection measurements by placing an ionic solution (0.5 M NaCl) in the feed compartment, an osmotic draw solution (2 M sucrose) in the permeate compartment, and measuring ion concentrations in both compartments as a function of time (Supplementary Fig. 16). Figure 2b (left axis) summarizes our results for 0.5 M NaCl rejection after 1 day for various LMs composed of NSNDs with pore diameters <10 nm, <25 nm, and <60 nm (referred to as porous MoS_2_s < 10, porous MoS_2_s < 25, and porous MoS_2_s < 60, respectively). For comparison, we also evaluated bare Anodisc alumina support and non-porous MoS_2_ NSLM in this study. While the supported Anodisc filter and non-porous MoS_2_ NSLM showed low rejection (<18%, consistent with the low rejection previously reported for MoS_2_ NSLMs)^21^, we find that introduction of porosity significantly enhanced the NaCl rejection compared to that of non-porous NSLMs. Porous MoS_2_s < 60 demonstrate a higher NaCl rejection (>57%), which improves to 80% for the porous MoS_2_s < 10 sample. Although the pore size is large compared to the Debye electrostatic screening length (<1 nm), we hypothesize that the creation of a larger number of flow pathways that traverse between charged pore edges and the underlying NSs lead to the improved salt rejection by hindering the transport of ions in preference to that of water. This behavior is also consistent with a prior study of graphene nanopores that indicates high ion selectivity in pores in the large regime (Debye length < pore radius), attributed to surface-charge-mediated cation selectivity^39^. Similarly, high salt rejection by nanoporous carbon composite membrane of a minimum pore size 30 nm was recently reported^40^. Finally, we find that incorporation of the NSND samples with the charged peptides has an even more dramatic impact on rejection, with ion rejection values reaching >99% at 0.5 M NaCl concentration (Fig. 2b). This improvement may be due to additional electrostatic interactions, increased in charge density in the interstitial area between sheets (evidenced by zeta potential measurements, see Supplementary Fig. 9), and also the tighter lamination of MoS_2_ NSs due to attractive forces between oppositely charged NSs. We hypothesize that membrane with two selective layers provides ultrahigh water/ion selectivity and more suitable for removing challenging impurities, such as small ions and organics^41,42^.

### Tuning pore size in NSND membranes

In addition to ion selectivity, water transport through the LMs is significantly affected by introduction of the pores and peptides (Supplementary Fig. 8). First, as a control, we find significant increases in water permeance (~4 times) upon incorporation of NDs (prepared using another method)^43,44^ into non-porous NS-LM, suggesting that NDs may play a role on introducing nanochannels within the LM that facilitate water transport. Further, as our expectation, porous MoS_2_ NSND-LMs with larger pore diameters have higher water permeance values than in smaller pore MoS_2_ NSND-LMs, suggesting that the extent of through-pathways within the LM shortens the path for water, resulting in higher water permeance values. The notable increase in water permeance rate by pores suggests that creation of through-pathways is dominant over the creation of channels by the NDs. Despite a decreased water permeance from (603 ± 38) to (432 ± 25) L m^−2^ h^−1^ bar^−1^ (LMH/bar) when going from <60 nm pores to <10 nm pores (Supplementary Fig. 8); we find that NSND-LMs prepared with both peptides present (for example, pep (+), pep (−) porous MoS_2_s < 10) maintain higher salt rejection (>99%) than NSND-LMs that contain exclusively positively charged peptides (~92%). The stronger interaction of the NSs (wrapped by both peptides) as confirmed by XRD measurement can be linked to the observed high salt rejection and the accompanying decrease in water flux, which can be attributed to charged interface and tighter assembly of the oppositely charged NSs by filling in any gaps or defects that may allow for salt transport. Based on these observations, we note that the overall LM structure, which is determined by interlayer alignment, surface charge, morphology, and defect structure, play a crucial role on water separation performance.

### Stability and performance of membranes

**Fig. 3:**
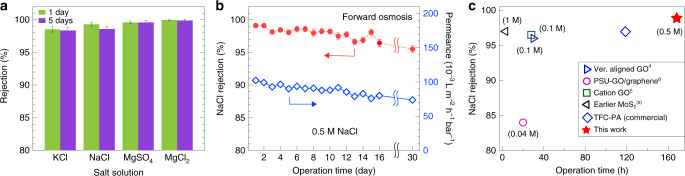
Salt rejection and stability of (pep (+), pep (−) porous MoS_2_s < 10) NSND-LMs under continuous operation. **a** Rejection values for various salts (0.5 M) by the membrane after 1 and 5 days of continuous operation using sucrose as a draw solution. **b** Rejection of NaCl (0.5 M) under continuous operation for >30 days (left axis) and water permeance during the continuous operation (right axis). Error bars, shown when larger than marker size, denote statistical reproducibility (*n* ≥ 3 for all measurements). **c** Comparison of various parameters related to performance of our membrane to other selected membrane materials^4–6,30^ (all measured using FO) and to commercial thin-film composite polyamide membrane tested under similar experimental conditions. The corresponding feed solution concentration is reported in the small brackets.

We also assessed the rejection of common salts in sea water by our (pep (+), pep (−) porous MoS_2_s < 10) NSND-LM after 1 and 5 days. As shown in Fig. 3a, rejection rates follow a steric effect, with ions of larger hydrated radii being rejected more efficiently (K^+^ < SO_4_^−2^ < Cl^−^ < Na^+^ < Mg^+2^)^45^. Given that water molecules form hydration shell to stabilize ions and that divalent ions hydrated shells are stronger than monovalent ones^4^, divalent ions are expected to experience larger barriers to enter into sub-nm voids within the NSND-LMs. For all 0.5 M salt solutions that we tested, we observed rejection values of >98%, even after 5 days of continuous operation. Further, we evaluated the rejection of NaCl as a function of concentration, and the membrane showed near complete (>99.99%) rejection below 0.5 M NaCl, even after 7 days of operation (Supplementary Fig. 13).

Since a major challenge with LMs is their mechanical and chemical stability under prolonged use, we performed continuous NaCl (0.5 M) salt-rejection experiments for 30 days (Fig. 3b). While we find a mild decrease in performance over this time, our membrane demonstrated a steady performance of >95% NaCl rejection throughout the experiment. Further, as shown in Fig. 3b (right axis), we observe stable water permeance during the continuous operation, which indicates little to no membrane clogging during prolonged use. The stable performance of our membrane can be attributed to tighter assembly formation due to stronger interlayer interaction of the oppositely charged NSs. As shown in Fig. 3c, these results compare favorably to the commercial TFC-PA SW30 membrane as well as recently reported functionalized and epoxy-encapsulated high-performance GO/graphene membranes. The water permeance value (5 L m^−2^ h^−1^, membrane thickness 1 µm) of our membrane is 6-, 10-, and 17-fold higher compared to the commercial (0.9 L m^−2^ h^−1^, tested in our set-up), functionalized GO (0.3 L m^−2^ h^−1^, membrane thickness 0.5 µm)^5^, and epoxy-encapsulated GO (0.5 L m^−2^ h^−1^, membrane thickness 5 µm)^4^ membranes, respectively.

**Fig. 4:**
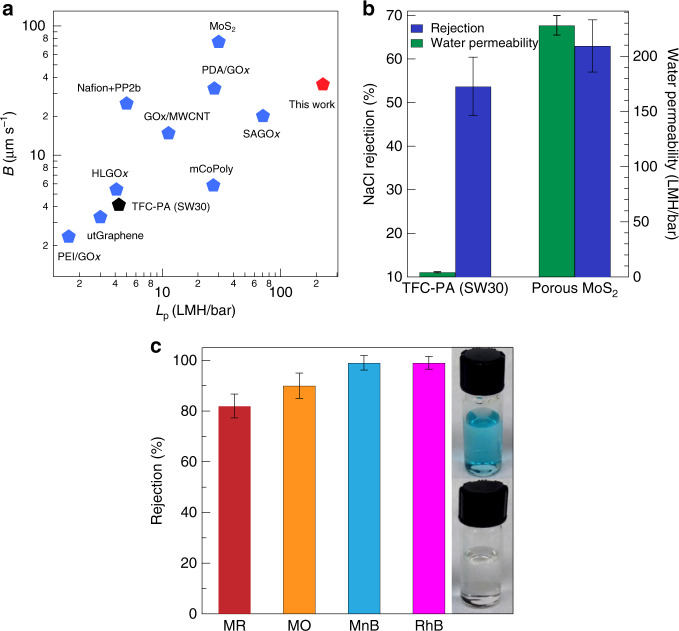
Comparison of (pep (+), pep (−) porous MoS_2_ s < 10) to other membranes and dye rejection data (measured in RO mode). **a** Log–log plot of salt permeability (*B*) and water permeability (*L*_p_) observed in commercial and various membranes reported in the literature (all measured in dead-end mode). Raw data and literature references are provided in Table S2. **b** NaCl (0.5 M) rejection (%) and water permeability of the commercial SW30-HR and porous MoS_2_ membrane. **c** Organic pollutant rejection measured using a 1-bar external pressure for methyl red (MR, electroneutral, *R*_H_ = 4.87 Å), methyl orange (MO, negative charge, *R*_H_ = 4.97 Å), methylene blue (MnB, positive charge, *R*_H_ = 5.04 Å), and rhodamine B (RhB, electroneutral, *R*_H_ = 6.15 Å). Hydrated radii are taken from ref. ^14^. Insets: Photographs of MB solution before and after filtration. Error bars denote statistical reproducibility (*n* ≥ 3 for all measurements).

*Comparison of RO performance*: Next, we performed salt (NaCl 0.5 M) rejection with the porous MoS_2_ membrane using RO mode in a dead-end filtration configuration set-up and compared the performance of our membranes with those of various membranes reported in the literature by using intrinsic properties, such as salt permeability (*B*) and water permeability (*L*_p_) of the membranes (Fig. 4a). We also tested commercial TFC-PA (SW30HR) RO membrane in our RO set-up under similar experimental conditions (black pentagon marker, Fig. 4a). It is noted that a lower rejection using dead-end mode (63 ± 12%) than using FO mode (>99%) was observed; such decrease in rejection performance in RO than in FO mode is also reported with extensively studied GO membranes^14,46–48^. Since the rejection of any membrane is defined as water/ion selectivity and mass transport processes, the applied pressure in dead-end mode is regarded to reduce selectivity by weakening the water–ion interaction while permeating through interlayer nanochannels^48^. Other key factors that contributed to the decrease in the overall salt rejection could be concentration polarization, nanochannel collapse^49^, and membrane compaction in the dead-end cell. Nevertheless, our membranes show superior performance, even with seawater-level feed salinities (for details, see Supplementary Table 2), which is typically very challenging for nanomaterial-based membranes.

*Chlorine susceptibility tests*: Low resistance to chlorine attack is a major bottleneck for RO water purification applications, because it precludes easy and cheap solutions to the problem of membrane biofouling. We benchmarked the chlorine resistance of our membranes (active chlorine concentration of 10,000 ppm) against commercial SW30HR membranes by measuring rejection and water flux at different time intervals (Supplementary Fig. 17). A continuous degradation of membrane performance was observed with the SW30 membrane upon chlorine exposure, indicated by a rapid reduction of rejection and increase in water permeability. MoS_2_ membrane was also affected by the exposure but much slower than the commercial SW30. To further test the chlorine susceptibility of porous MoS_2_ and SW30HR membranes, we measured their masses before and after immersing them in sodium hypochlorite solution for a 1-h period. While SW30HR commercial membrane loses 5.8% of its mass, MoS_2_ membrane loses only 3.4% under the same conditions. This loss in mass of MoS_2_ membrane is not surprising, as hypochlorite solution is a strong oxidizer and MoS_2_ itself is prone to oxidation^50,51^. A recent study revealed that hypochlorite etching in MoS_2_ starts from edges with dangling bond and extends toward center, while morphology and thickness remain reaction inert when those edges are covered^51^. Another study reported that hypochlorite does not induce morphological change of MoS_2_, rather increased surface charge due to adsorption of negatively charged hypochlorite onto its surface^52^. However, losing 3.4% of the mass of MoS_2_ is insufficient to cause drastic performance losses. SEM images of porous MoS_2_ membranes before and after exposure to chorine are shown in Supplementary Fig. 18.

### Filtration of organic contaminants

Finally, we investigated the selectivity of the porous MoS_2_ membrane for organic pollutants with different charges and hydrated radii (Fig. 4c). Filtration of organic pollutants methyl red (MR, electroneutral), methyl orange (negative charge), methylene blue (MnB, positive charge), and rhodamine B (RhB, electroneutral) were performed using a 1-bar external pressure, followed by ultraviolet–visible spectroscopic analysis of the feed and permeate solutions to evaluate the removal efficiency of the membrane. The membrane showed nearly 100% rejection per passage for both the neutral and charged organics with a hydrated radius >5 Å. We note that MnB, MR, and RhB are common industrial dyes used for coloring cotton, silk, and wool and thus are recognized as industrial and municipal water pollutants. Unlike a previously reported electrochemical separation process^53^, our method does not require multi-stage purification steps, allows higher water permeance, and could be energetically efficient. Membrane fouling in pressure-driven process is an inevitable challenge^54^. As a preliminary assessment of the fouling behavior of the membrane, we chose bovine serum albumin (BSA; 0.5 g L^−1^) as a model foulant and performed a loop filtration process using a 1-bar external pressure (see Supplementary Section 3 for details). The average water flux for pure water (232 ± 8.92) LMH/bar decreased slightly to (198 ± 14.8) LMH/bar for foulant solution. The calculated average flux recovery value was 96 ± 2%, which can be attributed to the charged smooth surface (surface roughness 1.88 nm, see Supplementary Fig. 11) as well as super hydrophilic nature of our membrane (see contact angle data in Supplementary Fig. 10 and Supplementary Movies).

**Fig. 5:**
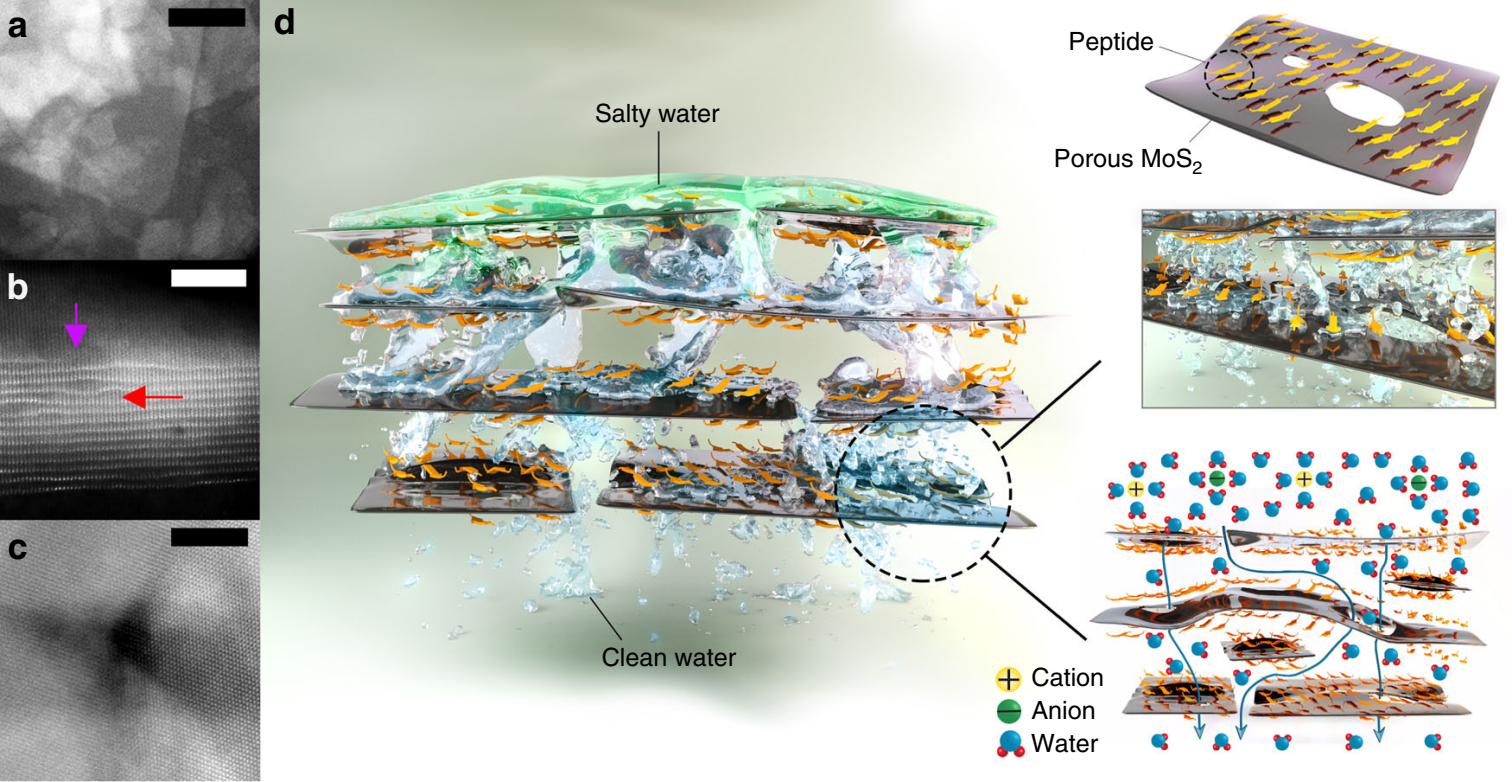
Sub-nanometer pathways for ion filtration in NSND-LMs. **a** Bird’s eye dark-field HAADF STEM image of an NSND laminate, showing NDs interspersed among NSs (scale bar = 50 nm, see also Supplementay Fig. 12). **b** A representative atomic HAADF STEM image of a thin peptide-modified NSND layer showing an average interlayer spacing of 7.8 ± 1.6 Å, a pocket of wider inter-sheet gaps presumably due to peptide intercalation shown by red arrow, and a void due to porous sheet structure shown by purple arrow (scale bar = 5 nm). **c** High-resolution HAADF STEM image of the peptide-decorated porous MoS_2_s < 10 NSs, showing a sub-nm pore (scale bar = 5 nm, see also Supplementay Fig. 4). **d** Cartoon and graphical depiction of the sub-nm pathways of water through the NSND-LM membranes. Blue arrows in the bottom right scheme highlight proposed trajectories of the paths of least resistance for water permeation, where ion (and dye) selectivity is achieved by exclusion due to steric and electrostatic ion–surface interactions.

Our overall results suggest that NSND-LMs provide both high throughput and efficient rejection of ions and small molecules due to the multiple pathways that water can permeate through the sub-nm voids in the highly porous laminate structure. A HAADF STEM image (Fig. 5a) shows numerous stacked NDs and small NSs seen as bright features. Figure 5b shows a high-resolution HAADF STEM image (cross-sectional and top view) of a thin peptide-modified NSND-LM. The undulating spacings between the laminated sheets (red arrow), presumably due to peptide occupancy, are in stark contrast with the image in Fig. 1d, which shows a relatively ordered spacing (apart from the stacking fault caused by the heterodimensional sheets and disk structure). In addition, voids caused by the porosity of the sheets are clearly seen in the image (purple arrow). We hypothesize that all of these features collectively dictate a multitude of sub-nanometer pathways by which water can transit through the membrane (Fig. 5d).

## Discussion

We report a straightforward and scalable cavitation method to create porous MoS_2_ NSs with controlled mean nanopore sizes. Further, we prepared composite LMs comprising the as-prepared porous MoS_2_ NSNDs composites and found that these exhibit ultrahigh water permeance values and very high ion selectivities, which can be dramatically improved with the introduction of self-assembling cationic and anionic peptides. We proposed a mechanism by which enhanced porosity within the membrane due to the presence of pores, finite sheet length, and NDs that act as spacers creates a highly porous yet charged interface that offers high permeance while achieving very high selectivity. Our membranes efficiently remove commonly found monovalent and divalent salts in seawater in the FO mode. They are also highly effective for removal of NaCl and a range of small organic molecules in RO mode. Moreover, our membranes withstand chlorine exposure and their extended operation times (month-scale) highlight their superior performance and durability as compared with the commercial and state-of-the-art laboratory membranes. Finally, we note that our methods for producing NSNDs, by virtue of its simplicity, is quite general and can be applied to other transition metal dichalcogenide materials, which will be the subject of future studies.

## Methods

### Materials

Molybdenum disulfide <2 µm powder was purchased at >98% purity (microlubrol), 1-methyl-2-pyrrolidone (NMP) was purchased from Fisher Scientific (ACS grade). All reagents were used as received. Peptides (white lyophilized powder) were purchased from Genscript (http://www.genscript.com) at >95% purity (high-performance liquid chromatography purified) and dissolved in ultrapure deionized (DI) water (Millipore, Billerica, MA) prior to use. Commercial TFC-PA RO membranes (SW30 HR) were purchased from Sterlitech (manufacturer Dow Filmtec). Sodium hypochlorite (NaOCl) solution was purchased from Sigma Aldrich.

### Synthesis of NS and NSNDs

One gram of natural MoS_2_ powder (cost ~$0.20 g^−1^) was added to 100 mL NMP solvent in a beaker and the mixture was bath-sonicated (Branson 2510 Ultrasonic) for 4 h (i.e., total delivered energy per unit volume 2880 J mL^−1^). After cooling down to room temperature, the exfoliated dispersion from top (80 mL) was further sonicated with a probe sonicator (Hielscher UIP 500 H) for 2 h (i.e., total delivered energy per unit volume 45,000 J mL^−1^). For the probe sonication step, the rotary regulator for pulse control was set to 1 (continuously switched on). The oscillation amplitude of the sonotrode was set to 100%, to ensure the most effective cavitation in the fluid that we believe leads to NS poration (formation, growth, and implosive collapse of vacuum bubbles in liquid). This method can be applied to other transition metal dichalcogenide material synthesis^55^.

### Tuning intrasheet pores in MoS_2_

Intrasheet pore diameter in the MoS_2_ NSs was tuned by altering a bath and probe sonication times and adjusting a rotary regulator of the probe sonicator as explained in the above synthesis method. Supplementary Table 1 provides the control parameters and the summary of our results.

### Preparation of peptide-decorated MoS_2_ colloids

To prepare stable suspensions of cationic and anionic MoS_2_, we utilized two different MoS_2_-binding peptides eight amino acids in length that alternate four positively charged lysine (K) or negatively charged glutamic acid (E) with hydrophobic residues phenylalanine (F), that is, KFKFKFKF and EFEFEFEF. These peptides self-assemble onto a MoS_2_ surface (Supplementary Fig. 7) to form a stable monolayer of β-tapes^56,57^ in which the cationic (KF)_4_ or anionic (EF)_4_ residues face outward toward the aqueous phase to promote dispersion of the coated NSs in water. The resulting coated MoS_2_ dispersions are highly stable in water for several months (Supplementary Fig. 6).

### AFM imaging of MoS_2_ and organized peptides on MoS_2_

AFM images of MoS_2_ NS were collected at ambient temperature using fast-scan dimension AFM (Bruker, USA) in tapping mode. Silicon cantilever were used (force constant 18 N m^−1^, resonance frequency 1400 kHz). To perform AFM imaging of peptide organization on MoS_2_ in liquid medium, the peptide was deposited in situ while imaging using ~200 µL of imaging buffer. All resulting samples were imaged with AFM (Dimension Icon, FastScan-type scan head) using a soft, sharp (*k* = 0.4 N m^−1^, nominal tip radius = 10 nm) cantilever in peak force imaging mode. While imaging the peptide, the peak force set point was <4 nN. Images were processed using the Nanoscope software.

### Characterization methods

SEM images were obtained by using the Hitachi S-4800 equipment. High-resolution TEM images were obtained using probe-corrected FEI Titan Themis STEM operated at 300 kV and using a HAADF detector. XRD measurements were performed (step size: 0.1, recording rate 0.1 s, current: 30 mA and voltage: 40 kV) using a Rigaku diffractometer with Cu Kα radiation (*λ* = 1.54 Å).

### Determination of MoS_2_ concentration

Concentration of the as-prepared MoS_2_ in the suspension was estimated by measuring mass of the MoS_2_ in the suspension obtained after passing the dispersion through an Anodisc alumina filter (Whatman, 0.02 µm pore size and a diameter 25 mm) and measuring the differential mass of the nanomaterial collected on the filter. For example, if 18 mg of the nanomaterial was collected on the filter by passing 3 mL of the suspension, the resulting concentration of the nanomaterial in the original suspension was 6 mg mL^−1^.

### Preparation of membranes

Freestanding membranes were prepared by vacuum filtration of the aqueous suspension of porous MoS_2_ or peptide-decorated porous MoS_2_ (3 mL, 6.2 ± 0.5 mg mL^−1^) through a porous Anodisc alumina filter (Whatman, 0.02 µm pore size and a diameter 25 mm; Sterlitech 0.02 µm pore size and a diameter 47 mm). In order to prepare (pep.+, pep.−) porous MoS_2_ membranes, positive peptide-decorated and negative peptide-decorated porous MoS_2_ was mixed well and left for 20 min for the reaction before forming membranes. As-prepared membranes were then baked in a vacuum oven at 50 °C for 12 h, which results in highly compact membranes with a slight disturbance in its uniformity. To regain smooth and homogenous membranes, 1 mL of the aqueous suspension of the porous MoS_2_ or peptide-decorated porous MoS_2_ were added by vacuum filtration and waited for a few hours for the membrane to completely dry prior to carrying out a water-separation experiment. We find that water-separation performance of the baked peptide–MoS_2_ composite membrane is much better than the baked MoS_2_ membrane, which could be due to a number of reasons: first, the enhanced charge afforded by the charged peptide; second, the tighter vertical assembly of the NSs promoted by the peptide spacing; third, mechanical integrity that is provided by the peptides^58^, which form extended networks with intermolecular interactions on top of the MoS_2_ surface.

### Membrane fouling test

The fouling behavior of a membrane essentially depends on several factors chemical and physical features of the membrane surface, such as pore morphology, pore size, pore charge, and most importantly the hydrophobicity^54^. The molecules of the organic foulant are likely to attach to the hydrophobic surface because of the hydrophobic–hydrophobic interaction, for example, graphene and synthetic polymers such as polyvinylidene fluoride, polysulfone, polyethersulfone, and polyacrylonitrile, are highly prone to organic and biological fouling due to hydrophobic–hydrophobic interactions. On the other hand, water molecules are likely to be adsorbed at the hydrophilic interface, thereby minimizing adsorption of organic foulants^59^. To evaluate the membrane dynamic fouling behavior, BSA (0.5 g L^−1^) was chosen as a model organic foulant. The loop filtration process performed consisted of three steps: filtration of pure water, filtration of BSA, and filtration of pure water after rinsing the membrane with pure water. The loop process was repeated for five cycles (times) to determine the flux recovery (FR), which was obtained by using the following equation:

1 $${\mathrm{FR}}\left( \% \right) = \left( {\frac{{J_i}}{J}} \right) \times 100({\mathrm{\% }})$$

where *J* is the initial flux of the membrane for pure water and *J*_*i*_ is the membrane flux for water at the end of each loop process (after rinsing the membrane with pure water) after cycle *i*. The calculated average flux recovery value was 96 ± 2%, which can be attributed to the possible hydrophilic nature of our membrane, as well as its surface charge and smoothness.

### Contact angle (CA) measurements

To understand the wettability of the membranes, water and NaCl solution CA measurements were performed by drop-casting DI water and 0.5 M NaCl on the surface of the membranes. To be consistent in all the measurements, the volume of the droplets is controlled to ~10 µL, and the photographs of the droplets were taken using a camera within 2 min of casting the droplets on the membrane surface (Supplementary Fig. 10). The measurements were performed at least in three different areas of the membrane and the average value of the CA is reported. The CA values for NaCl were slightly less than water CA values for all the membranes except Anodisc support (Supplementary Fig. 10), which can be linked to the interactions between charged ionic solution and the charged surface of the membranes.

### Calculation of permeability and salt rejection

Permeability of the membrane was calculated using the following relation:

2 $${\mathrm{Permeability}} = \frac{{V_{\mathrm{p}}}}{{t.A.\nabla P}}$$

where *V*_p_ is the permeate volume, *t* is the permeation time, *A* is the effective area of the membrane, and ∇*P* is the applied pressure.

Rejection (NaCl and dye) of the membrane in RO mode was calculated by:

3 $${\mathrm{Rejection}}\left( \% \right) = \left( {1 - \frac{{C_{\mathrm{p}}}}{{C_{\mathrm{f}}}}} \right) \times 100(\% )$$

where *C*_p_ and *C*_f_ are the concentrations of salt or probe molecule in the permeate and the feed solution, respectively.

For several days of continuous operation in FO mode where one needs to add salt solution in the feed compartment and extract filtrate solution from the permeate compartment, salt rejection can be calculated by using the following relation:

4 $$R = \left( {1 - \frac{{\left( {C_{\rm{p}} + \Delta C_{\rm{p}}} \right) \times \left( {V_{\rm{p}} + \Delta V} \right) - C_{\rm{p}}V_{\rm{p}}}}{{C_{\rm{f}}\Delta V}}} \right)$$

where

Δ*C*_p_ = increase in the concentration of salt in the permeate side when its volume goes from *V*_p_ to (*V*_p_ + Δ*V*);

*V*_p_ = initial volume in the permeate side;

Δ*V* = increase in volume in the permeate side;

*C*_f_ = concentration of salt in the feed side;

$\left( {C_{\rm{p}} + \Delta C_{\rm{p}}} \right) \times \left( {V_{\rm{p}} + \Delta V} \right)$ is the final amount of salt on permeate side;

*C*_p_*V*_p_ is the initial amount of salt on permeate side; and

*C*_f_Δ*V* is the amount of salt that would have gone through in the case of zero rejection.

If the condition *C*_p_ ≪ (1 − *R*)*C*_f_ is satisfied, then the expression simplifies to:

5 $$R = \left( {1 - \frac{{V_{\rm{p}}\Delta C_{\rm{p}}}}{{C_{\rm{f}}\Delta V}}} \right)$$

This follows from comparing the *V*_p_Δ*C*_p_ and *C*_p_Δ*V* terms. From Eq. (4)

$\left( {C_{\rm{p}} + \Delta C_{\rm{p}}} \right) \times \left( {V_{\rm{p}} + \Delta V} \right) - C_{\rm{p}}V_{\rm{p}} = \left( {1 - R} \right)C_{\rm{f}}\Delta V$. For small Δ*V*, neglecting the second-order term, we get

$V_{\rm{p}}\Delta C_{\rm{p}} + C_{\rm{p}}\Delta V = \left( {1 - R} \right)C_{\rm{f}}\Delta V$, which gives $\,\,\quad\Delta C_{\rm{p}} = \left[ {\left( {1 - R} \right)C_{\rm{f}} - C_{\rm{p}}} \right]\frac{{\Delta V}}{{V_{\rm{p}}}}$. Hence,

$$\frac{{C_{\rm{p}}\Delta V}}{{V_{\rm{p}}\Delta C_{\rm{p}}}} = \frac{{C_{\rm{p}}\Delta V}}{{\left[ {\left( {1 {\,}-{\,} R} \right)C_{\rm{f}} {\,}-{\,} C_{\rm{p}}} \right]\Delta V}} = \frac{{C_{\rm{p}}/\left( {1 {\,}-{\,} R} \right)C_{\rm{f}}}}{{1 {\,}-{\,} C_{\rm{p}}/\left( {1 {\,}-{\,} R} \right)C_{\rm{f}}}} \ll 1$$ for $$\frac{{C_{\rm{p}}}}{{\left( {1 {\,}-{\,} R} \right)C_{\rm{f}}}} \ll 1$$.

## Supplementary information

SI

Contact Angles 1

Contact Angles 2

## Data Availability

Data supporting the findings in this manuscript are available from the corresponding author upon request.

## References

[1] Werber JR, Osuji CO, Elimelech M (2016). Materials for next-generation desalination and water purification membranes. Nat. Rev. Mater..

[2] Elimelech M, Phillip WA (2011). The future of seawater desalination: energy, technology, and the environment. Science.

[3] Jain T (2015). Heterogeneous sub-continuum ionic transport in statistically isolated graphene nanopores. Nat. Nanotechnol..

[4] Abraham J (2017). Tunable sieving of ions using graphene oxide membranes. Nat. Nanotechnol..

[5] Chen L (2017). Ion sieving in graphene oxide membranes via cationic control of interlayer spacing. Nature.

[6] Morelos-Gomez A (2017). Effective NaCl and dye rejection of hybrid graphene oxide/graphene layered membranes. Nat. Nanotechnol..

[7] Surwade SP (2015). Water desalination using nanoporous single-layer graphene. Nat. Nanotechnol..

[8] Fischbein MD, Drndić M (2008). Electron beam nanosculpting of suspended graphene sheets. Appl. Phys. Lett..

[9] Koenig SP, Wang L, Pellegrino J, Bunch JS (2012). Selective molecular sieving through porous graphene. Nat. Nanotechnol..

[10] Radha B (2016). Molecular transport through capillaries made with atomic-scale precision. Nature.

[11] Hong S (2017). Scalable graphene-based membranes for ionic sieving with ultrahigh charge selectivity. Nano Lett..

[12] Feng J (2016). Observation of ionic Coulomb blockade in nanopores. Nat. Mater..

[13] Hu M, Mi B (2013). Enabling graphene oxide nanosheets as water separation membranes. Environ. Sci. Technol..

[14] Akbari A (2016). Large-area graphene-based nanofiltration membranes by shear alignment of discotic nematic liquid crystals of graphene oxide. Nat. Commun..

[15] Yang Q (2017). Ultrathin graphene-based membrane with precise molecular sieving and ultrafast solvent permeation. Nat. Mater..

[16] Ritt, C., Werber, J. R., Deshmukh, A. & Elimelech, M. Monte Carlo simulations of framework defects in layered two-dimensional nanomaterial desalination membranes: implications for permeability and selectivity. *Environ. Sci. Technol*. **53**, 6214–6224 (2019). 10.1021/acs.est.8b06880 31066551

[17] Zhou K-G (2018). Electrically controlled water permeation through graphene oxide membranes. Nature.

[18] Yeh C-N, Raidongia K, Shao J, Yang Q-H, Huang J (2015). On the origin of the stability of graphene oxide membranes in water. Nat. Chem..

[19] Loh KP, Bao Q, Eda G, Chhowalla M (2010). Graphene oxide as a chemically tunable platform for optical applications. Nat. Chem..

[20] Zheng S, Tu Q, Urban JJ, Li S, Mi B (2017). Swelling of graphene oxide membranes in aqueous solution: characterization of interlayer spacing and insight into water transport mechanisms. ACS Nano.

[21] Wang Z (2017). Understanding the aqueous stability and filtration capability of MoS_2_ membranes. Nano Lett..

[22] Sun P, Wang K, Zhu H (2016). Recent developments in graphene-based membranes: structure, mass‐transport mechanism and potential applications. Adv. Mater..

[23] Deng M, Kwac K, Li M, Jung Y, Park HG (2017). Stability, molecular sieving, and ion diffusion selectivity of a lamellar membrane from two-dimensional molybdenum disulfide. Nano Lett..

[24] Jiang D-e, Cooper VR, Dai S (2009). Porous graphene as the ultimate membrane for gas separation. Nano Lett..

[25] Cohen-Tanugi D, Grossman JC (2012). Water desalination across nanoporous graphene. Nano Lett..

[26] Heiranian, M., Farimani, A. B. & Aluru, N. R. Water desalination with a single-layer MoS_2_ nanopore. *Nat. Commun*. **6**, 8616 (2015). 10.1038/ncomms9616 PMC4634321 26465062

[27] Li W, Yang Y, Weber JK, Zhang G, Zhou R (2016). Tunable, strain-controlled nanoporous MoS_2_ filter for water desalination. ACS Nano.

[28] Feng J (2016). Single-layer MoS_2_ nanopores as nanopower generators. Nature.

[29] Farimani AB, Min K, Aluru NR (2014). DNA base detection using a single-layer MoS_2_. ACS Nano.

[30] Hirunpinyopas W (2017). Desalination and nanofiltration through functionalized laminar MoS2 membranes. ACS Nano.

[31] Yoon, S.-H. *Membrane Bioreactor Processes: Principles and Applications* (CRC, 2015).

[32] Mi B (2019). Scaling up nanoporous graphene membranes. Science.

[33] Zhang Y, Qian Z, Ji B, Wu Y (2016). A review of microscopic interactions between cavitation bubbles and particles in silt-laden flow. Renew. Sustain. Energy Rev..

[34] Shchukin DG, Skorb E, Belova V, Möhwald H (2011). Ultrasonic cavitation at solid surfaces. Adv. Mater..

[35] Suslick, K. In *Kirk-Othmer Encyclopedia of Chemical Technology* 5th edn Vol. 26 (ed. Kirk-Othmer, R. E.) 517–541 (Wiley, 1998).

[36] Sapkota, B. & M. Wanunu. Porous transition metal dichalcogenides nanosheets and quantum dots fabrication thereof. US patent 62/536,228 (2017).

[37] Joshi R (2014). Precise and ultrafast molecular sieving through graphene oxide membranes. Science.

[38] Rasamani KD, Alimohammadi F, Sun Y (2017). Interlayer-expanded MoS_2_. Mater. Today.

[39] Rollings RC, Kuan AT, Golovchenko JA (2016). Ion selectivity of graphene nanopores. Nat. Commun..

[40] Chen W (2018). High-flux water desalination with interfacial salt sieving effect in nanoporous carbon composite membranes. Nat. Nanotechnol..

[41] Esfandiar A (2017). Size effect in ion transport through angstrom-scale slits. Science.

[42] Werber JR, Elimelech M (2018). Permselectivity limits of biomimetic desalination membranes. Sci. Adv..

[43] Sapkota B (2017). Peptide-decorated tunable-fluorescence graphene quantum dots. ACS Appl. Mater. Interfaces.

[44] Sapkota, B., Mustata, M., Zhang, J., Grigoryan, G. & Wanunu, M. J. DNA-binding properties of peptide-functionalized graphene quantum dots. *Biophys. J*. **108**, 393a (2015).

[45] Wang L (2017). Fundamental transport mechanisms, fabrication and potential applications of nanoporous atomically thin membranes. Nat. Nanotechnol..

[46] Wang J (2016). Graphene oxide as an effective barrier on a porous nanofibrous membrane for water treatment. ACS Appl. Mater. Interfaces.

[47] Lyu J (2018). Separation and purification using GO and r-GO membranes. RSC Adv..

[48] Sun P (2016). Intrinsic high water/ion selectivity of graphene oxide lamellar membranes in concentration gradient-driven diffusion. Chem. Sci..

[49] Huang H (2013). Ultrafast viscous water flow through nanostrand-channelled graphene oxide membranes. Nat. Commun..

[50] Lutsik V, Sobolev A (2005). The investigation of the kinetics of hydrochemical oxidation of metal sulphides with the aim of determination of the optimal conditions for the selective extraction of molybdenum from ores. J. Min. Metall. B Metall..

[51] Zhang P (2019). Chemically activated MoS_2_ for efficient hydrogen production. Nano Energy.

[52] Wang Y (2018). Water-soluble MoS_2_ quantum dots are a viable fluorescent probe for hypochlorite. Microchim. Acta.

[53] Mao X (2018). Energetically efficient electrochemically tunable affinity separation using multicomponent polymeric nanostructures for water treatment. Energy Environ. Sci..

[54] van der Marel P (2010). Influence of membrane properties on fouling in submerged membrane bioreactors. J. Membr. Sci..

[55] Pandey LK, Sapkota B, Wanunu MJ (2020). Ions exclusion by the bio-inspired WS2 lamellar membrane under different driving forces. Biophys. J..

[56] Mustata G-M (2016). Graphene symmetry amplified by designed peptide self-assembly. Biophys. J..

[57] Sapkota B, Pandey L, Benabbas A, Wanunu MJ (2019). Highly-stable bio-inspired peptide/MoS2 membranes for efficient water desalination. Biophys. J..

[58] Wanunu, M. & Sapkota, B. Porous membranes comprising nanosheets and fabrication thereof. US patent application 20190039028 (2019).

[59] Hegab HM, Wimalasiri Y, Ginic-Markovic M, Zou L (2015). Improving the fouling resistance of brackish water membranes via surface modification with graphene oxide functionalized chitosan. Desalination.

