# Author Correction: High permeability sub-nanometre sieve composite MoS_2_ membranes

**DOI:** 10.1038/s41467-020-17422-y

**Published:** 2020-07-21

**Authors:** Bedanga Sapkota, Wentao Liang, Armin VahidMohammadi, Rohit Karnik, Aleksandr Noy, Meni Wanunu

**Affiliations:** 1 Department of Physics, Northeastern University, Boston, MA 02115 USA; 2 Kostas Advanced Nanocharacterization Facility (KANCF), Northeastern University, Burlington, MA 01803 USA; 3 Department of Materials Engineering, Auburn University, Auburn, AL 36849 USA; 4 Department of Mechanical Engineering, Massachusetts Institute of Technology, 77 Massachusetts Avenue, Cambridge, MA 02139 USA; 5 Physical and Life Sciences Directorate, Lawrence Livermore National Laboratory, 7000 East Avenue, Livermore, CA 94550 USA; 6 University of California Merced, School of Natural Sciences, Merced, CA 95343 USA

**Keywords:** Two-dimensional materials, Nanopores

Correction to: *Nature Communications* 10.1038/s41467-020-16577-y, published online 2 June 2020.

The original version of this Article contained an error in Fig. 2b, in which the *y*-axis was inadvertently omitted.

