# Supplementary material for: High permeability sub-nanometre sieve composite MoS2 membranes
Source: Nat Commun. 2020 Jun 2;11:2747. doi: 10.1038/s41467-020-16577-y (PMC7265532; doi:10.1038/s41467-020-16577-y)
Supplement: Supplementary file 1 — SI [file 41467_2020_16577_MOESM1_ESM.pdf]

# Supplementary Information

# High Permeability Sub-Nanometre Sieve Composite $MoS_2$ Membranes

*Bedanga Sapkota[1], Wentao Liang[2], Armin VahidMohammadi[3], Rohit Karnik[4], Aleksandr Noy[5], and Meni Wanunu[1]**

[1]Department of Physics, Northeastern University, Boston, Massachusetts 02115, USA

[2]Kostas Advanced Nanocharacterization Facility (KANCF), Northeastern University, Burlington, Massachusetts 01803, USA

[3]Department of Materials Engineering, Auburn University, Auburn, AL 36849, USA.

[4]Department of Mechanical Engineering, Massachusetts Institute of Technology, 77 Massachusetts Avenue, Cambridge, Massachusetts 02139, USA

[5]Biology and Biotechnology Division, Physical and Life Sciences Directorate, Lawrence Livermore National Laboratory, Livermore, CA 94550, USA; School of Natural Sciences, University of California Merced, Merced, California 95343, USA

*Corresponding author: wanunu@neu.edu

## Table of contents

1. Supplementary Methods ……………………………………………………………………......3
2. Forward and reverse osmosis…………………………………………………………...4
3. Supplementary Figures ……………………………………………………………........5
4. Supplementary Table.………………………………………………………….................13
5. Set-up for water separation experiments using external pressure....……………………….....18
6. Set-up for osmotic pressure-driven ions sieving experiments……………………………...19
7. Conductivity probe calibration….……………………………………………………......21
8. Chlorine exposure evaluation…………………………………………………………….21
9. Supplementary References …………………………………………………………………23

## SUPPLEMENTARY METHODS

**Supplementary Table 1:** Control parameters to tune pore as well as nanosheet diameter.

| Category | Bath sonication (hr.) | Probe sonication (hr.) | Pore diameter (nm)[a] | Mean NS diameter (nm) | NS layers |
|---|---|---|---|---|---|
| A | 4 | - | No pores | 352±44 | tri-tetralayer |
| B | 4 | 2 | <60 | 163±20 | single-bilayer |
| C | 4 | 1 | <45 | 285±46 | single-bilayer |
| D | 3 | 1 | <10 | 785±83 | single-bilayer |
| E | 2 | 1 | <10 | 1090±101 | single-trilayer |
| F | 1 | 2 | <25 | 1472±176 | bi-trilayer |

[a] Pore size in the $MoS_2$ sheets was estimated from AFM measurements.

Although single- to multi-pores were observed in the individual smaller diameter nanosheets, the density of pores per-nanosheet significantly increases in the relatively bigger diameter nanosheets. For example, we observed an average of 30 pores (pore size <10 nm) in nanosheets of average size ~ 1 µm (Supplementary Figure 3a). It is noted that the number of pores could be significantly higher, because the detection of smaller size pores <5 nm in diameter along with nanosheet of average diameter ~ 1 µm is beyond the resolution limit of our AFM (average tip radius ~ 10 nm). While high-resolution scanning TEM image of the same sample shows pores as small as ~ 1 nm (Supplementary Figure 4), characterizing a large set of pores in a NS using HR-TEM is prohibitively expensive and impractical.

**Preparation of NDs:**

To prepare nanodisks (NDs), the exfoliated dispersion obtained in Category B (after probe sonication) was left undisturbed at least for 2 days. The supernatant was collected and further treated using bath sonication for a 1 h. The average diameter of the NDs was 38±14 nm.

**Purification and transfer of porous NS/NDs from NMP to water:**

Transfer of porous NSs/NDs from NMP to water was carried out in two steps: first from NMP to methanol, and second from methanol to water. In the first step, 20 mL of methanol was added to 10 mL of the exfoliated dispersion of NSs/NDs in NMP and the resulting dispersion was centrifuged at 5,000 rpm for 30 min. This was repeated 3-4 times to remove multilayer $MoS_2$ flakes. Finally, the resultant product was dialyzed in a low-cutoff dialysis bag (Spectra/Por Biotec Cellulose Ester, 100−500 Da) against methanol for 15 hours to remove unwanted small molecules. After dialysis-based purification, the dispersion of porous NSs/NDs in methanol was added dropwise to deionized water with gentle stirring using a steel needle. The vial was kept in an open-to-air dust-free environment to allow methanol to evaporate while stirring gently with a steel needle every 20 min for a few hours. The process was continued until a dark dispersion of $MoS_2$ in water was observed.

# SUPPLEMENTARY DISCUSSION

## Forward and reverse osmosis:

Because of the low energy consumption, easy cleaning process, low fouling, and high salt rejection, forward osmosis (FO) is considered to be an attractive emerging technology for desalination applications[1]. FO desalination uses water-soluble salt or other molecules (highly concentrated solution) to generate osmotic pressure, which draws fresh water molecules spontaneously across a semi-permeable membrane from the low concentrated salt solution (feed solution). Here, we performed FO desalination by filling equal volume (10 mL) of sucrose (2 M) and NaCl or other salts solution (0.5 M) in the permeate and feed compartment (see Supplementary Figure 16), respectively, which was separated by the $MoS_2$ membrane (effective area 0.51 $cm^2$). Magnetic stirring was applied in both feed and permeate compartments to avoid possible concentration gradients. Osmotic pressure (Π) of an aqueous solution can be determined by using Van't Hoff relation:

$$\Pi = \Phi i R T M \qquad (1)$$

where $M$ is the molar concentration (mol/L), $R$ is the gas constant (0.08206 L atm·$mol^{-1}$·$K^{-1}$), $T$ is the temperature in Kelvin, $\Phi$ is osmotic coefficient ($\Phi_{NaCl}$=0.93 and $\Phi_{sucrose}$=1.02), and ί is the number of ions or molecules into which the dissolved species dissociate ($ί_{NaCl}$ = 2 and $ί_{sucrose}$ = 1). With all these values, equation (1) leads to an osmotic pressure gradient of ∼ 28 bar, which pulls water molecules from the feed compartment to the permeate compartment. Salt (NaCl) rejection by the membrane under continuous operation is estimated by using equation (5). The conductivity of the salt solutions was measured using an InPro conductivity sensor (Mettler Toledo). Further, to test the mechanical robustness of the membranes, we carried out filtration of NaCl (0.5 M) for a prolonged period (> 30 days) and calculated the NaCl rejection after every 24 hours using equation (5). The observed 1.5 mL increase in permeate column with (pep (+), pep (-) porous $MoS_2$s<10)-NSND-LM over 6 hours of period corresponds to a water flux of 5 $Lm^{-2}h^{-1}$, which is lower than reverse osmosis, however remarkable for forward osmosis[2]. Our membrane showed 10-fold higher water flux (5 $Lm^{-2}h^{-1}$) than recently reported epoxy-encapsulated GO-Gr membrane (0.5 $Lm^{-2}h^{-1}$)[3] and 17-fold higher flux than cationic control GO membrane (0.3 $Lm^{-2}h^{-1}$)[4].

Reverse osmosis experiments were performed using the Sterlitech HP4750 stirred cell. We also performed organics filtration experiments using a home-made permeation cell (Supplementary Figure 15), and found similar results to that of the Sterlitech cell.

## SUPPLEMENTARY FIGURES

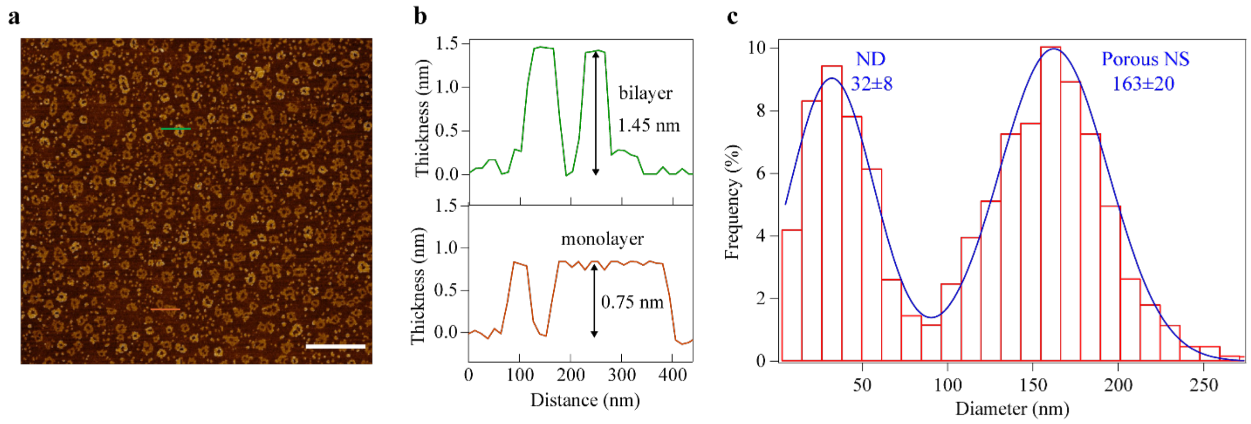


**Supplementary Figure 1: Quantification of porous $MoS_2$ NSs and NDs (Category B, Table S1). a,** Topographic atomic force micrographs of as-prepared porous $MoS_2$s<60 NSs dispersed on freshly-cleaved mica. The dark features in the middle of the NSs are pores formed from knockout of material from within the NS, whereas the ejected ND products are seen as dots in the image. Scale bar = 500 nm. **b,** Thickness profile of the porous NSs as shown by green and orange lines in the topographic image. Most of the NSs are single layer with a few bilayers (higher contrast NSs in the image). **c,** Diameter distribution of the NDs and NSs.

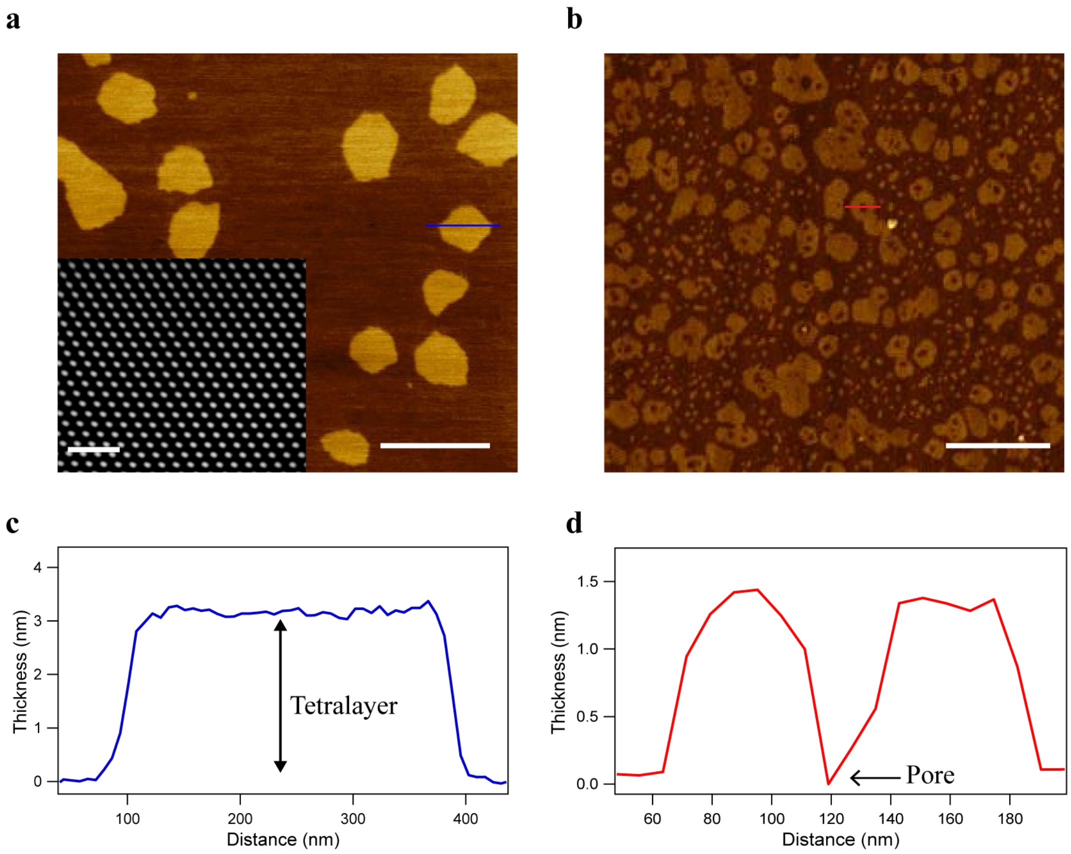


**Supplementary Figure 2: Characterization of non-porous (Category A, Table S1) and porous-$MoS_2$s<45 (Category C, Table S1) NSs. a,** Topographic atomic force micrographs of non-porous $MoS_2$ NSs dispersed on freshly-cleaved mica (see Table S1). Inset: Aberration-corrected scanning transmission electron microscopy (STEM) image of a $MoS_2$ lattice (Scale bar = 1 nm). **b,** Topographic atomic force micrographs of as-prepared porous $MoS_2$s<45 NSs dispersed on freshly-cleaved mica. The dark features in the NSs are pores formed from knockout of material from within the NS, whereas the ejected ND products are seen as dots in the image. Scale bars = 500 nm for both AFM images. **c,** Thickness profile of the non-porous NS as shown by blue line in the topographic image, showing tetralayer nanosheet. **d,** Thickness profile of the porous NS as shown by red line in the topographic image, down spikes in the line profile is due to pore in the nanosheet. In this preparation, most of the NSs are bilayer with a few monolayers.

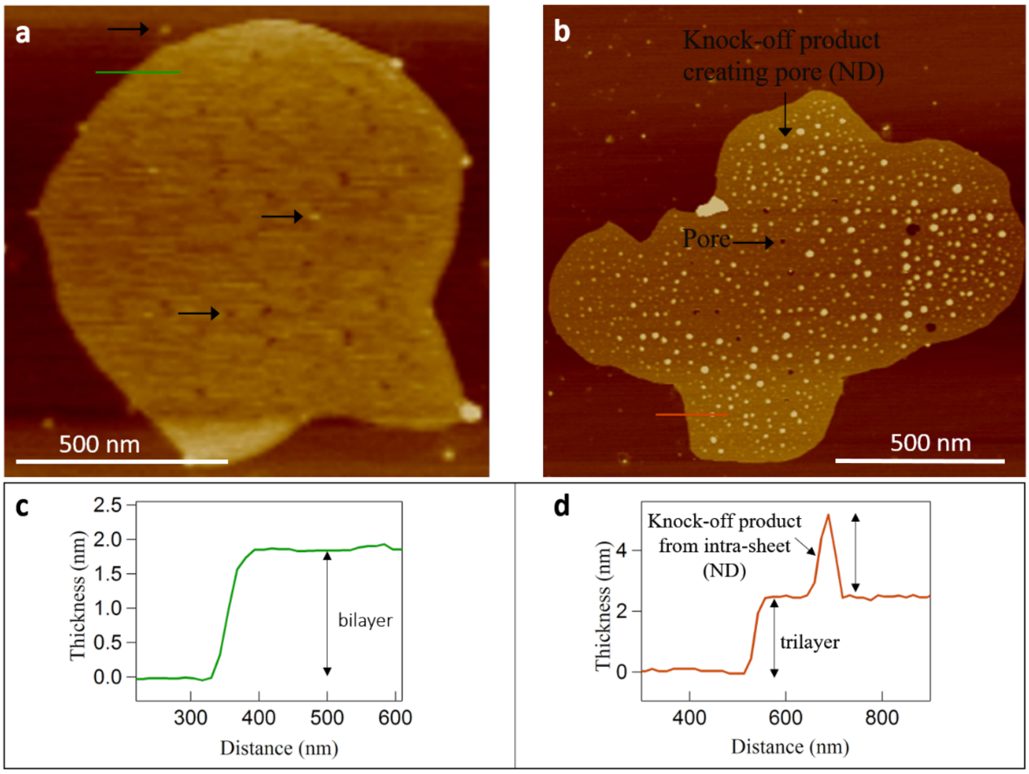


**Supplementary Figure 3: AFM images confirm proof-of-concept of size-controllable synthesis of NSs as well as intra-sheet pores (Category E and F, Table S1). a,** Topographic image of porous $MoS_2s$<10 NS of ~ 1 µm on freshly-cleaved mica, black arrows show pore as well as knock-off products from intra-sheet, which are of similar diameter as of the pores. **b,** Topographic image of porous $MoS_2s$<25 NS of ~ 1.4 µm, where intra-sheet hole and knock-off products (NDs) are shown by black arrows. **c,** Thickness profile of the green line shown in panel a, which shows that the porous NS is a bilayer. **d,** Thickness profile of the orange line shown in the figure b, which shows the thickness of the knock-off product (NDs) is a trilayer, which is same in thickness of the porous NS.

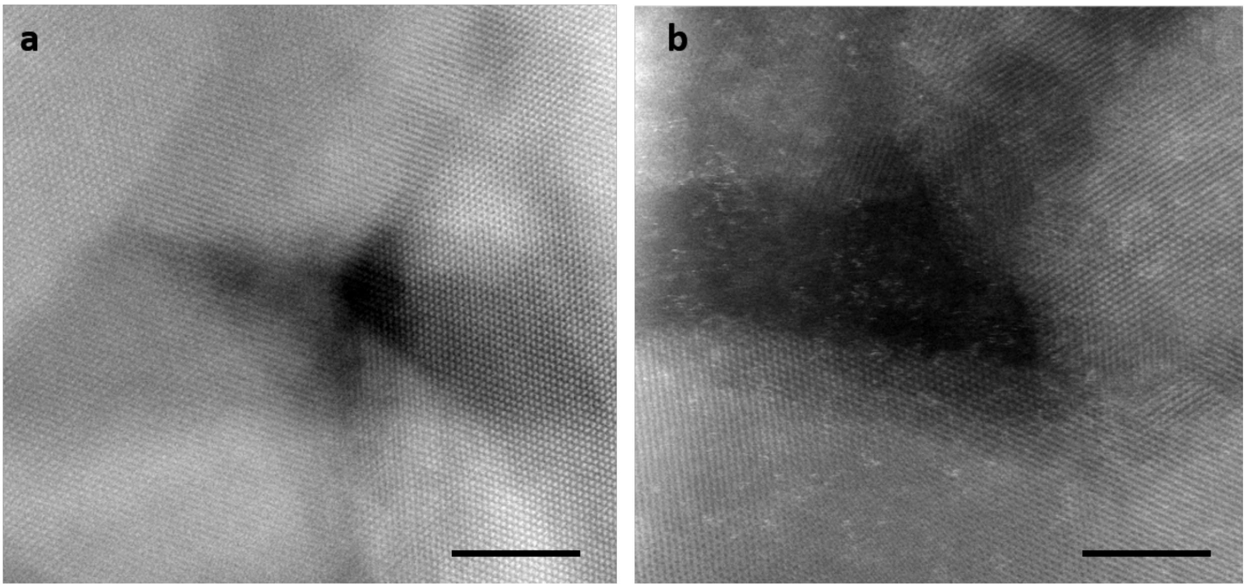


**Supplementary Figure 4: Characterization of the porous $MoS_2$ nanosheets**. **a, b,** High-resolution TEM images of the peptide-decorated porous $MoS_2$s<10 NSs, showing sub-nm and ~5 nm pores. Scale bars = 5 nm.

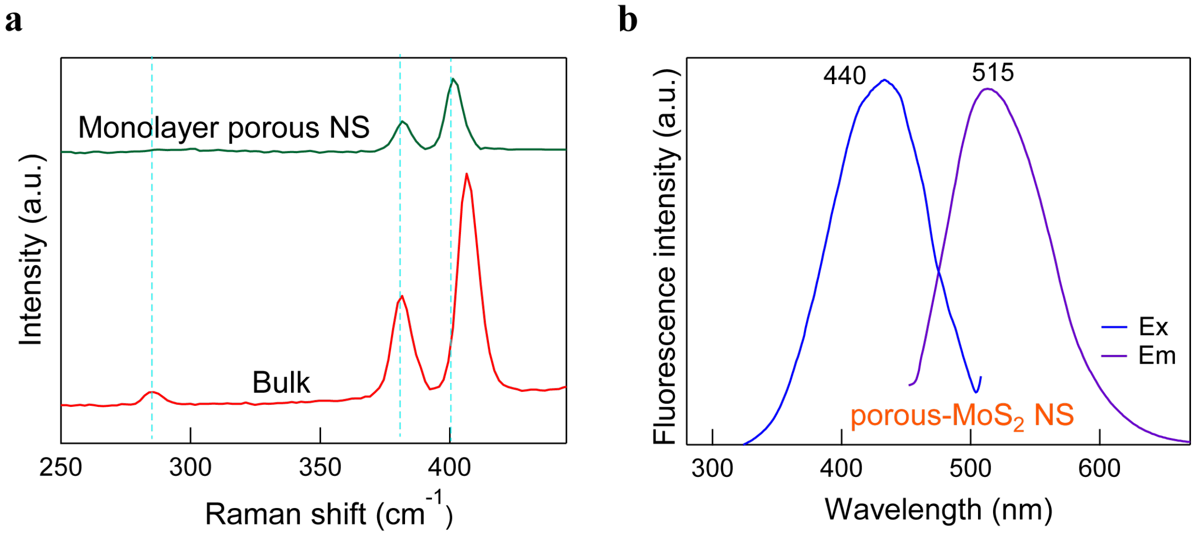


**Supplementary Figure 5: Raman and Fluorescence measurements. a,** Raman spectra of bulk $MoS_2$ and porous-NS, peak around 286 $cm^{-1}$ is absent for monolayer porous-$MoS_2$. Further, the separation between the two higher-shift modes is 19.53 $cm^{-1}$, which confirms single-layer $MoS_2$ NSs. **b,** Excitation and emission spectra of porous-$MoS_2$ NS (excitation wavelength used for emission spectra and emission wavelength used for excitation spectra are indicated above spectra).

**a** **b**

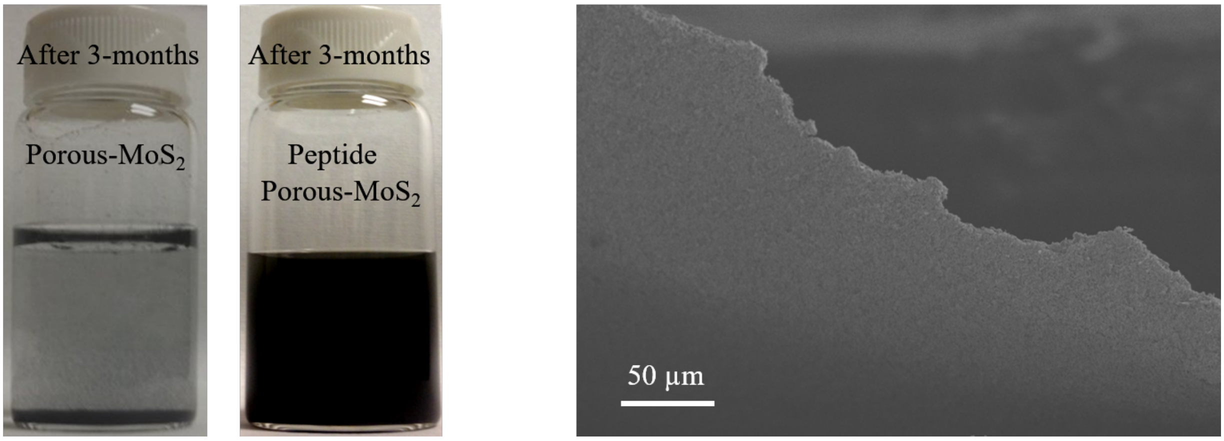


**Supplementary Figure 6: Excellent dispersity upon peptide-decoration and the freestanding membrane. a,** Photograph of porous $MoS_2$ in water (12.6 mg/mL) before (left) and upon positive peptide-decoration (right), both after 3 months, suggest that stability is induced by hydrophilic peptide decoration. **b,** An SEM image of a freestanding membrane prepared from the positively charged and negatively-charged porous $MoS_2$ NSs (also see Schematic S1).

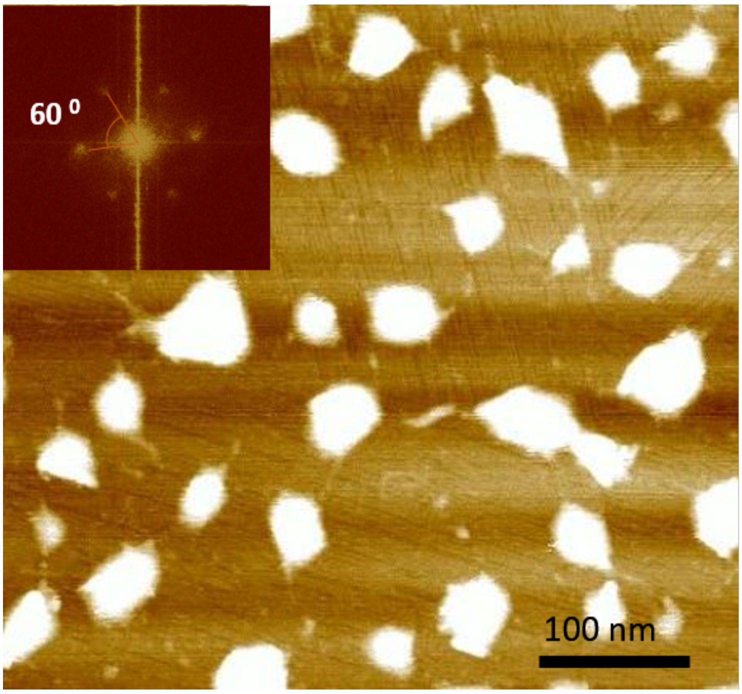


**Supplementary Figure 7: Peptide self-assembly onto $MoS_2$ surface.** AFM image taken in water shows a self-assembled peptide design-peptide film on the $MoS_2$ surface. White spots are probably due to water adsorption by the hydrophilic peptide surface, and ordered lines represent beta-tapes formed from peptide assembly on the $MoS_2$ surface. Inset is FFT of the image, which shows the three possible orientations of the peptide on the surface.

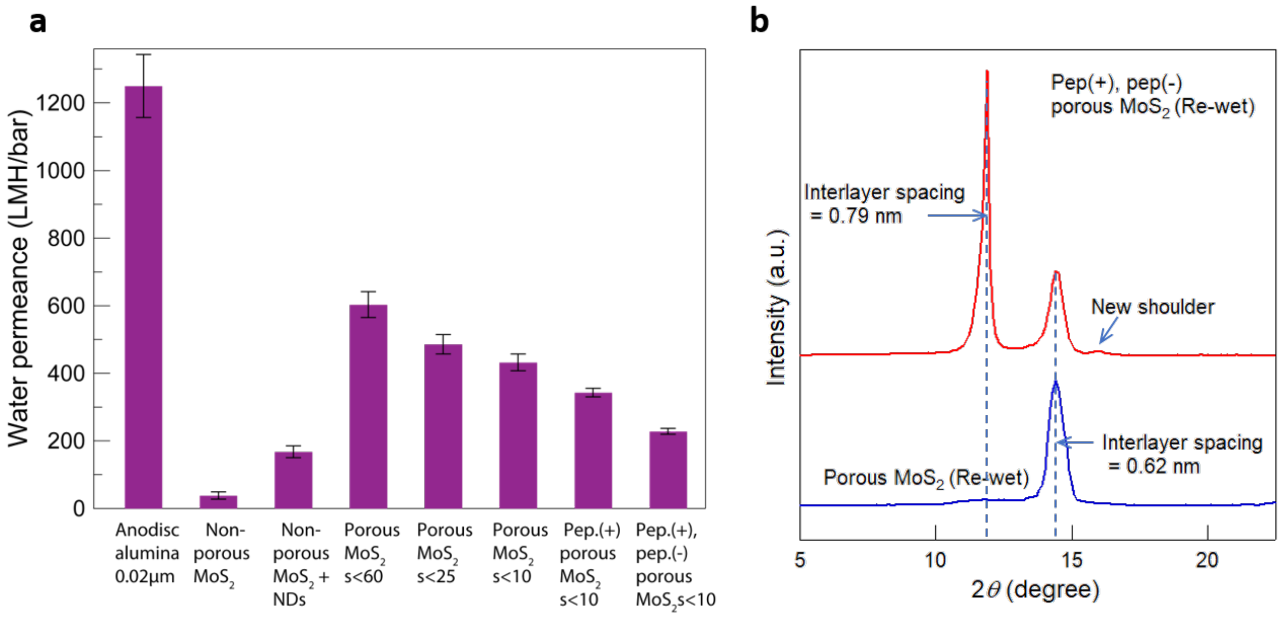


**Supplementary Figure 8: Effect of interlayer alignment, pore size, nanodisk spacing, and peptide presence on water permeance. a,** Comparison of water permeance by a bare Anodisc alumina filter, a non-porous $MoS_2$ NSL, a non-porous $MoS_2$ NSL with NDs, a porous $MoS_2$ NSL of different pore diameters, and a peptide-decorated (pep. +, pep. -) porous $MoS_2$ NSL. Water permeance was measured using a 1 bar external pressure difference ($n \geq 3$ for all measurements). **b,** X-ray diffraction for porous $MoS_2$ and a peptide-decorated (pep. +, pep. -) porous $MoS_2$ re-wet membranes showing 0.17 nm increased in interlayer spacing upon peptide incorporation. A new shoulder is also appeared at an angle of 16.1° with the peptide-decorated (pep. +, pep. -) porous $MoS_2$ membrane.

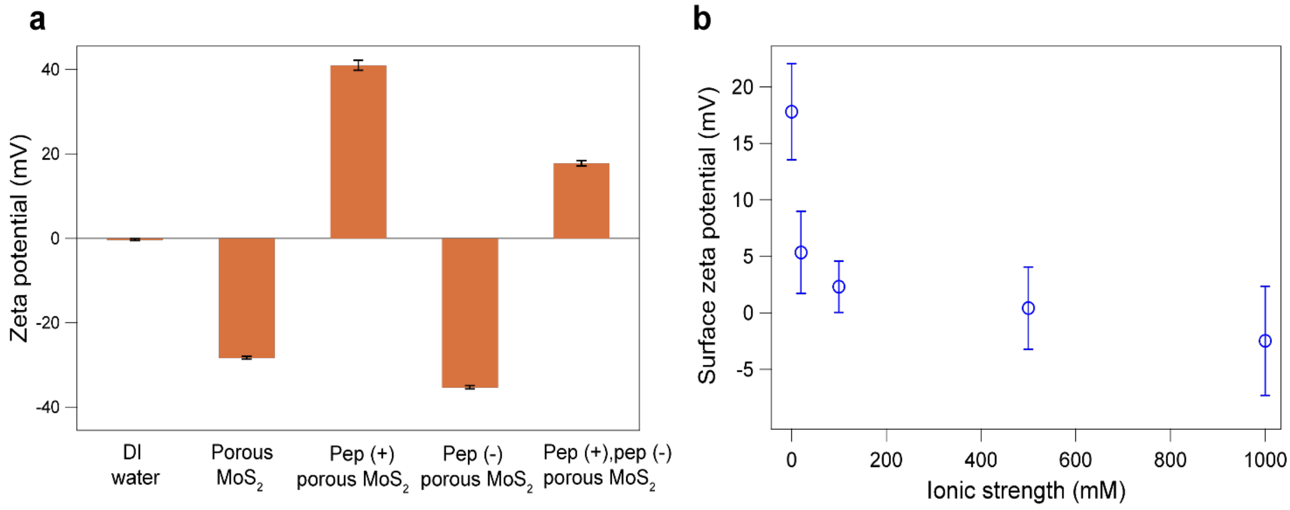


**Supplementary Figure 9: Zeta potential and surface zeta potential measurements. a,** Zeta potential of DI water, and aqueous suspension of porous $MoS_2$, positive peptide-decorated porous $MoS_2$, negative peptide-decorated porous $MoS_2$, and the mixture of both (mixture of equal concentration and volume of positively and negatively-charged porous $MoS_2$). **b,** The surface zeta potential of (pep. (+), pep. (-)) porous $MoS_2$ as a function of ionic strength (NaCl).

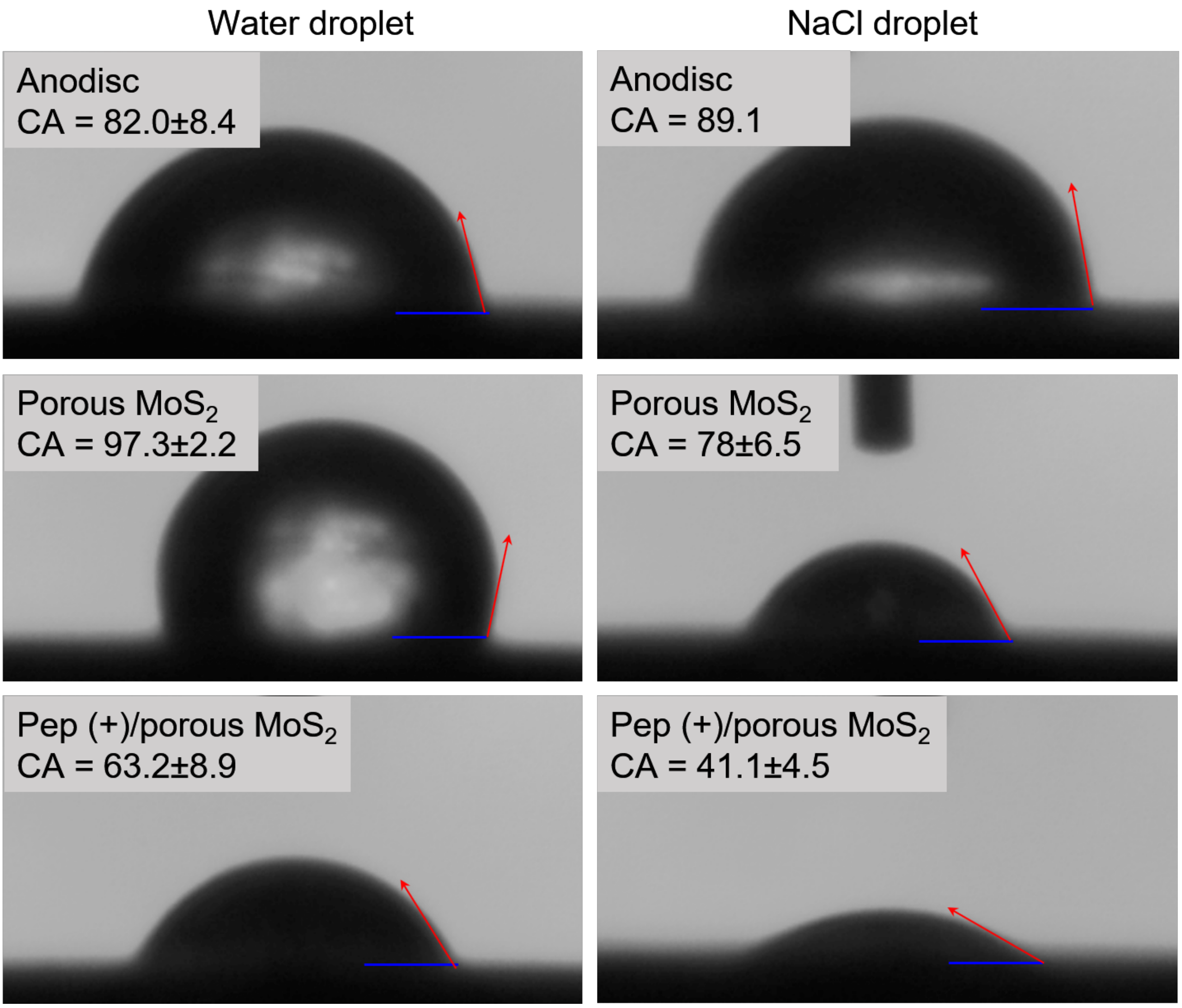


**Supplementary Figure 10: Water and NaCl contact angle (CA) measurements.** Photographs of water droplet (left) and NaCl droplet (right) on Anodisc support, as well as on the porous $MoS_2$ and positive peptide-decorated porous $MoS_2$ membranes. Contact angle of water and NaCl droplets on the (pep. (+), pep. (-)) porous $MoS_2$ cannot be measured due to super hydrophilic nature of the membrane surface (videos are provided).

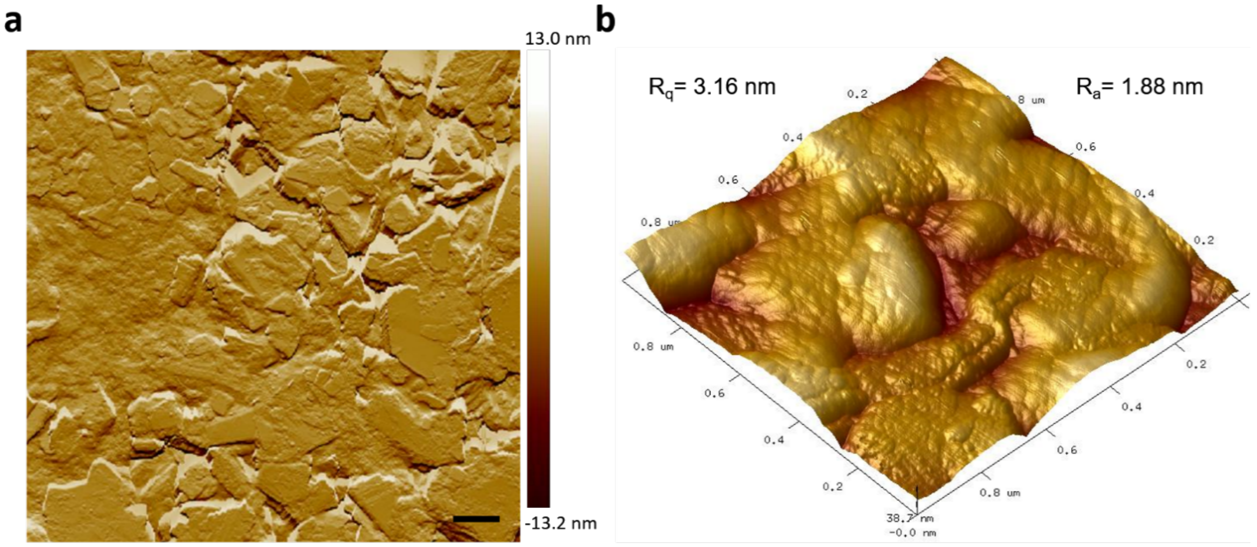


**Supplementary Figure 11: AFM images of (pep (+), pep (-) porous $MoS_2$s<10)-LM. a,** AFM height image of (pep. (+), pep. (-)) porous $MoS_2$ membrane showing the smooth surface and NSs assembly. **b,** 3D rendering of the small portion of the same image with the surface roughness values. Scale bar = 500 nm for panel a.

# SUPPLEMENTARY TABLE

**Supplementary Table 2:** Comparison of various parameters related to performance of our NSND membrane with other selected membrane materials in RO mode.

| Membrane | Mode | Membrane thickness | Water permeance (LMH/bar) | Feed NaCl concentration (mM) | NaCl Rejection (%) | Reference |
|---|---|---|---|---|---|---|
| HLGO | Dead-end | 8 nm | ~5 | 1000 | ~10 | 5 |
| Shear aligned GO | Dead-end | 150 nm | 71±5 | 34 | 33 | 6 |
| Nafion+PP2b | Dead-end | 17.5 µm | 3 | 7 | 43 | 7 |
| PEI+GO | Dead-end | n/a | 1.62 | 20 | 42 | 8 |
| PDA-coated GO | Dead-end | ~16 nm | 27.6 | 20 | 19 | 9 |
| commercial polyamide | Cross-flow | n/a | 15.4 | 98 | 55 | 10 |
| modified co-polymer | Dead-end | 150 µm | 27 | 10 | 62 | 11 |
| GO/MWCNT | Dead-end | 40 nm | 11.3 | 10 | 39 | 12 |
| ultrathin graphene | Dead-end | 53 nm | 3.3 | 20 | 42 | 13 |
| Earlier $MoS_2$ | Dead-end | 1 µm | 30 | 20 | 10 | 14 |
| TFC-PA (SW30)[a] | Dead-end | n/a | 4.3 | 500 | 53.7±13.4 | - |
| **(Cationic+anionic) porous $MoS_2$-s10** | **Dead-end** | **1 µm** | **228** | **500** | **63±12** | **This work** |

[a]Commercial TFC-PA (SW30 HR) membranes were purchased from Sterlitech and tested in our system under the similar experimental conditions.

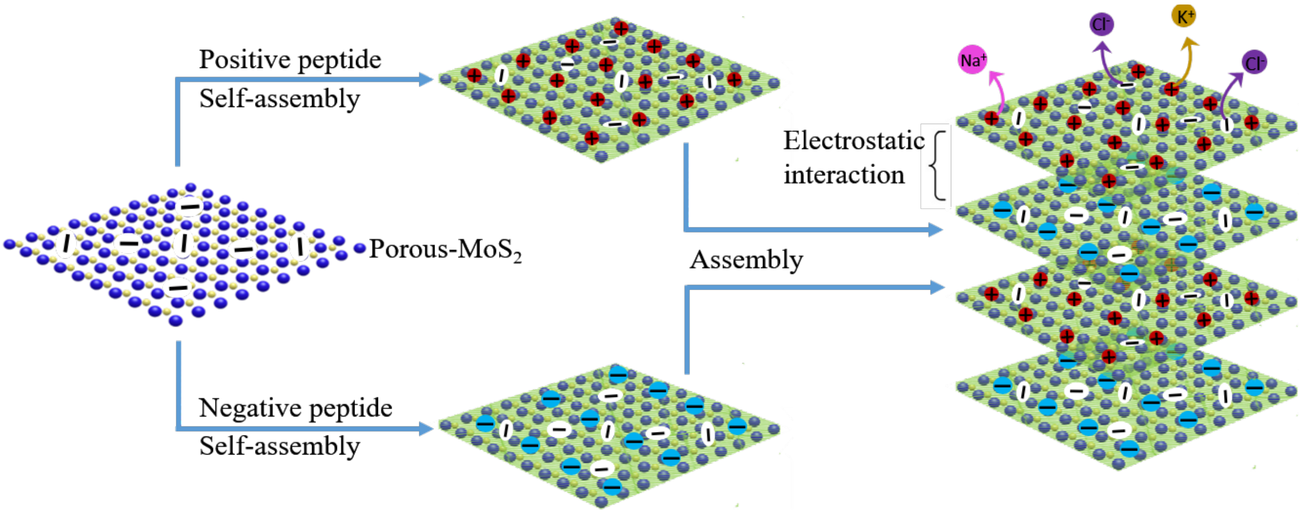


**Supplementary Schematic 1:** Schematic representation showing possible assembly of oppositely-charged porous $MoS_2$ nanosheets. In the schematic, the Mo and S atoms of $MoS_2$ are represented by blue and yellow spheres, whereas self-assembly of the designed peptides onto $MoS_2$ surface is represented by green lines. The alternating positive/negative membrane surface will repel ions, whereas the negatively-charged pore periphery repels anions. A synergistic effect of this combination accounts for the significant improvement in salt rejection.

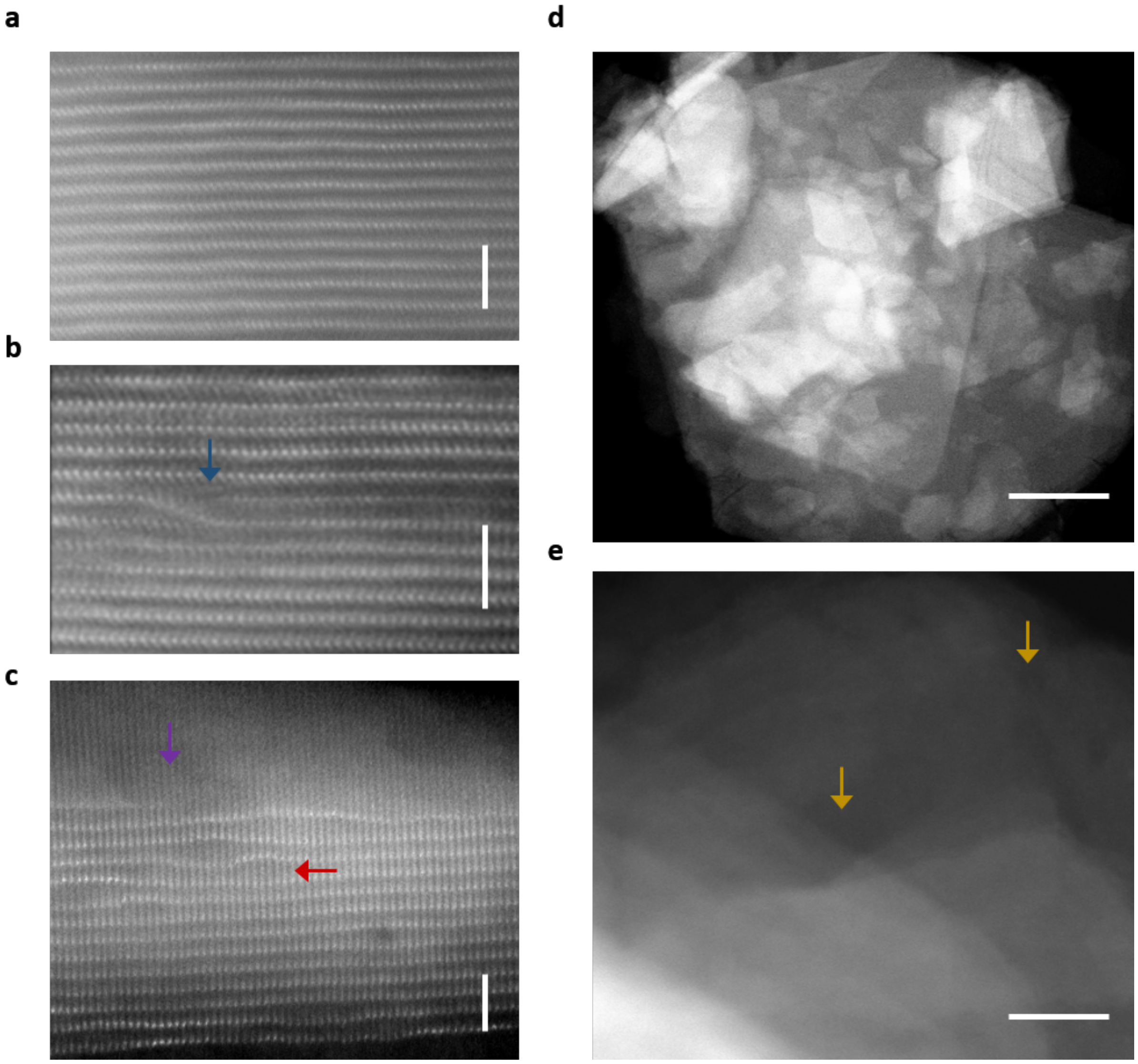


**Supplementary Figure 12: High-resolution imaging of non-porous, porous and peptide-decorated porous membranes**. **a,** High-resolution TEM image of a thin laminate cross-section of non-porous $MoS_2$ NSs, showing highly-ordered layered structure of average interlayer spacing 6.2 Å. **b,** High-resolution TEM image of a thin laminate cross-section of porous $MoS_2$ NSNDs, showing stacking faults induced by nanosheet porosity and intersheet/ND stacking defects. A defect that results in interlayer voids, as shown by blue arrow. **c,** Dark-field scanning TEM image of a thin peptide-modified porous NSND layer, with intersheet slits due to peptide intercalation shown as red arrow and voids due to porous sheet structure shown as purple arrow. The average interlayer spacing is 7.8±1.6 Å (Scale bars = 2 nm). Image is 512x512 pixels, taken at 300kV at 3.6Mx magnification using a HAADF detector, pixel dwell time of 40µs. **d,** birds-eye dark-field STEM image of a NSND laminate, showing interspersed NDs and NSs as brighter features (scale bar = 50 nm). **e,** High-magnification STEM image of the laminate, with voids due to porous sheet structure shown as yellow arrows (Scale bar = 10 nm).

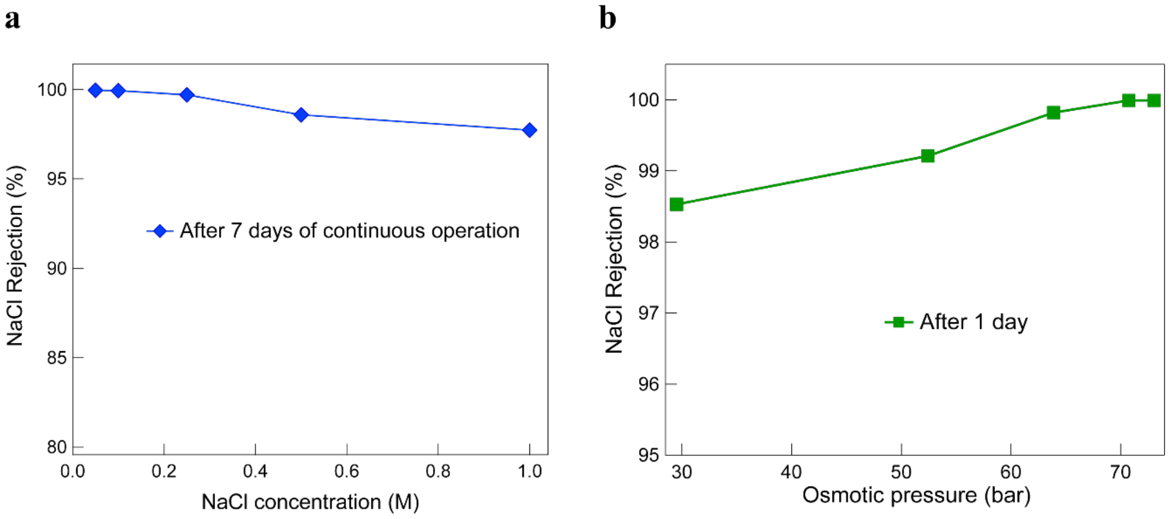


**Supplementary Figure 13: NaCl rejection as a function of concentration by (pep (+), pep (-) porous $MoS_2$s<10)-NSND-LM.** **a,** NaCl rejection of the membrane against concentration, showing 100% rejection below 0.3 M even after 7 days of continuous operation. **b,** NaCl rejection as a function of osmotic pressure after 1 day of operation.

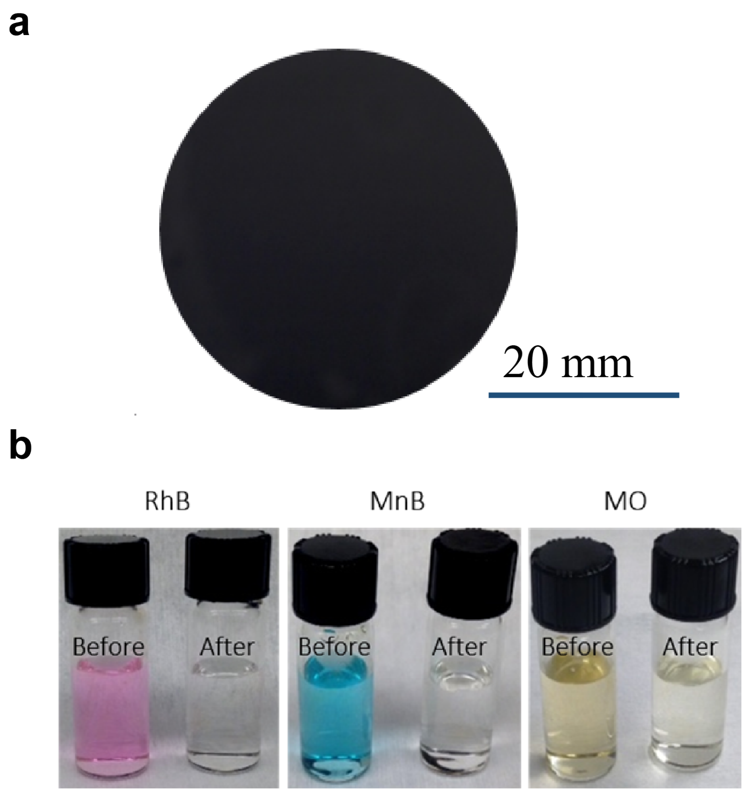


**Supplementary Figure 14: Porous $MoS_2$ membrane and photographs of various dye solutions before and after filtration. a,** Optical image of the porous $MoS_2$ membrane. **b,** Photographs of Rhodamine B (RhB), methylene blue (MnB), and methyl orange (MO) before and after filtration through (pep (+), pep (-) porous $MoS_2$s<10)-NSND-LM.

## 1. Set-up for water separation experiments using external pressure

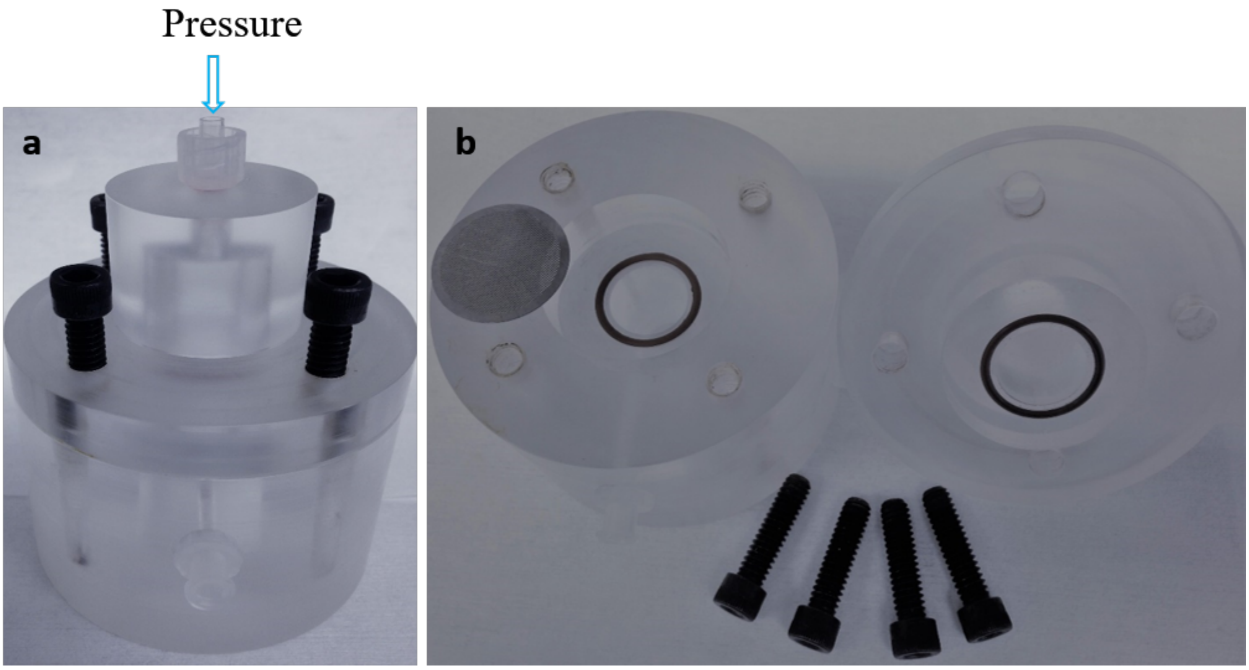


**Supplementary Figure 15: Permeation cell setup for external pressure driven water permeance. a,** Photograph of acrylic cell used for pressure-driven water permeance experiments. Feed compartment is shown on top, and permeate compartment is shown on bottom. Pressure was applied to feed compartment (shown by arrow) by connecting a hose to a regulated $N_2$ tank. Membranes were kept between two O-rings and fixed using four screws to provide a good seal. **b,** Inside view that shows O-ring arrangement, as well as a 25-mm porous supporting metal disc to mechanically support the Anodisc alumina filter (top left).

## 2. Set-up for osmotic pressure-driven ions sieving experiments

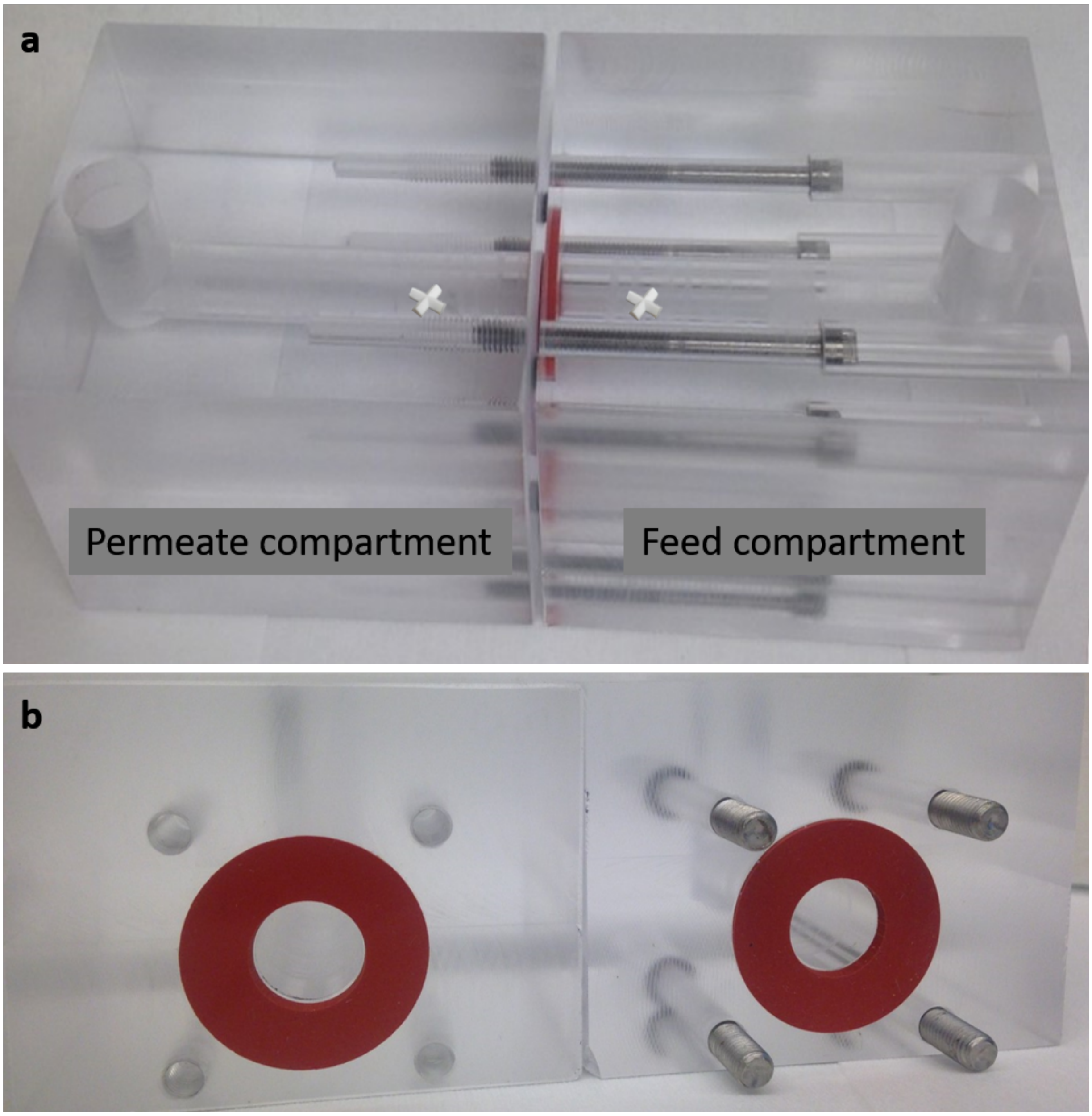


**Supplementary Figure 16: Permeation cell. a,** Experimental setup showing acrylic-based feed and permeate compartments used for ions sieving experiments. Membranes were kept between two silicone washers and fixed using four screws to provide a leakproof environment. Magnetic stirring was applied in both feed and permeate compartments to avoid possible concentration gradients during experiment. **b,** Inside view of feed and permeate compartments that shows silicone washers arrangement along with four screws.

# SUPPLEMENTARY NOTES

## Calculation of permeability and salt rejection

Permeability of the membrane was calculated using the following relation:

$$\text{Permeability} = \frac{V_p}{t.A.\nabla P} \quad (2)$$

Where $V_p$ is the permeate volume, t is the permeation time, A is the effective area of the membrane and $\nabla P$ is the applied pressure.

Rejection (NaCl and dye) of the membrane in RO mode was calculated by:

$$\text{Rejection (\%)} = \left(1 - \frac{C_p}{C_f}\right) \times 100(\%) \quad (3)$$

where $C_p$ and $C_f$ are the concentrations of salt or probe molecule in the permeate and the feed solution, respectively.

For several days of continuous operation in FO mode where one needs to add salt solution in the feed compartment and extract filtrate solution from the permeate compartment, salt rejection can be calculated by using the following relation:

$$R = \left(1 - \frac{(C_p + \Delta C_P) \times (V_p + \Delta V) - C_p V_p}{C_F \Delta V}\right) \quad (4)$$

Where

$\Delta C_P$= increase in the concentration of salt in the permeate side when its volume goes from $V_p$ to $(V_p + \Delta V)$

$V_p$= Initial volume in the permeate side

$\Delta V$= increase in volume in the permeate side

$C_F$= concentration of salt in the feed side

$(C_p + \Delta C_P) \times (V_p + \Delta V)$ is the final amount of salt on permeate side

$C_p V_p$ is the initial amount of salt on permeate side

$C_F \Delta V$ is the amount of salt that would have gone through in the case of zero rejection

If the condition $C_p \ll (1 - R)C_F$ is satisfied, then the expression simplifies to:

$$R = \left(1 - \frac{V_p \Delta C_P}{C_F \Delta V}\right) \quad (5)$$

This follows from comparing the $V_p \Delta C_P$ and $C_p \Delta V$ terms. From Eq. (4) $(C_p + \Delta C_P) \times (V_p + \Delta V) - C_p V_p = (1 - R)C_F \Delta V$ . For small $\Delta V$, neglecting the 2nd order term, we get

$V_p \Delta C_P + C_p \Delta V = (1 - R)C_F \Delta V$, which gives $\Delta C_P = [(1 - R)C_F - C_p]\frac{\Delta V}{V_p}$. Hence,

$$\frac{C_p \Delta V}{V_p \Delta C_p} = \frac{C_p \Delta V}{[(1-R)C_F - C_p]\Delta V} = \frac{C_p/(1-R)C_F}{1 - C_p/(1-R)C_F} \ll 1 \text{ for } \frac{C_p}{(1-R)C_F} \ll 1.$$

## Conductivity probe calibration:

To determine the concentration of salt in the permeate side, the conductivity probe was calibrated for each salt solution using their known concentrations. Since the forward osmosis experiment was performed using sucrose as a draw solution, the calibration was conducted in sucrose solution of same concentration while varying a range of salt concentrations. An empirical quadratic regression was carried out to determine the calibration coefficients from the obtained data using the following relation:

$$C = a_o + a_1\,\sigma + a_2\sigma^2 \tag{6}$$

where $C$ is molar concentration of salt solution, $\sigma$ is the measured conductivity in mS/cm, and the quadratic regression coefficients $a_o$ and $b_o$were determined by fitting the calibration equation.

## Chlorine exposure evaluation:

Effect of chlorination on membrane performance was carried out by soaking porous $MoS_2$ and commercial polyamide thin film composite (TFC) RO membranes in an aqueous solution of sodium hypochlorite (solution pH 7.5, active chlorine 10,000 ppm) at 22 $^{o}$C for different time interval. The soaked membranes were then thoroughly rinsed with DI water before performing the RO experiments. To quantify chlorine exposure intensity, product of total chlorine concentration and soaking time concept was employed, resulting (ppm×h) notation. The chlorine-resistance performance of the membranes was evaluated by normalizing salt rejection and water flux as follows:

$$R_n = R/R_o \text{ and } F_n = F/F_o$$

where $R_o$ and $F_o$ are the initial rejection and flux, whereas R and F are the instantaneous rejection and flux at different times.

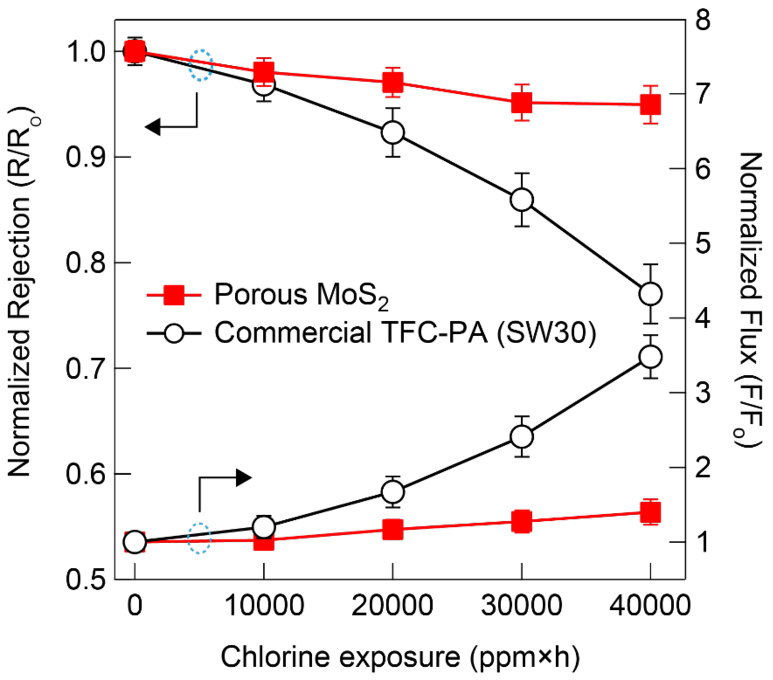


**Supplementary Figure 17: Chlorine susceptibility of porous $MoS_2$ and commercial membrane.** Chlorine exposure evaluation of commercial SW30-HR and the porous $MoS_2$ membrane.

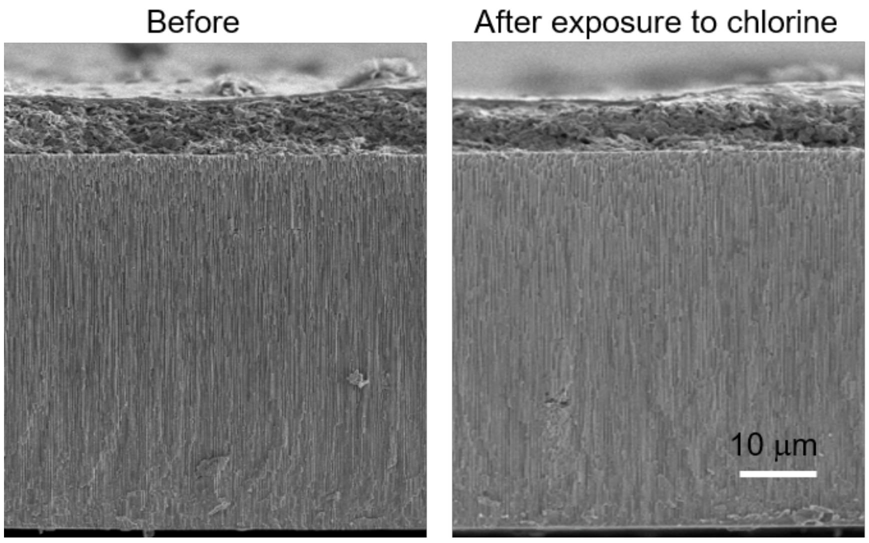


**Supplementary Figure 18: Chlorine susceptibility of porous $MoS_2$ membrane.** SEM images of porous $MoS_2$ membranes before (left image) and after exposure (right image) to sodium hypochlorite (10,000 ppm) for 2 hrs. Under 10% $MoS_2$ thickness change was observed, due to $MoS_2$ oxidation and/or possibly a collapse of the membrane layer.

## Supplementary References


1 Zhao, S., Zou, L., Tang, C. Y. & Mulcahy, D. Recent developments in forward osmosis: opportunities and challenges. *Journal of membrane science* **396**, 1-21 (2012).

2 Chekli, L. *et al.* A comprehensive review of hybrid forward osmosis systems: Performance, applications and future prospects. *Journal of Membrane Science* **497**, 430-449 (2016).

3 Abraham, J. *et al.* Tunable sieving of ions using graphene oxide membranes. *Nature Nanotechnology* **12**, 546-550 (2017).

4 Chen, L. *et al.* Ion sieving in graphene oxide membranes via cationic control of interlayer spacing. *Nature* **550**, 380-383 (2017).

5 Yang, Q. *et al.* Ultrathin graphene-based membrane with precise molecular sieving and ultrafast solvent permeation. *Nature materials* **16**, 1198 (2017).

6 Akbari, A. *et al.* Large-area graphene-based nanofiltration membranes by shear alignment of discotic nematic liquid crystals of graphene oxide. *Nature communications* **7**, 10891 (2016).

7 Cohen, E. *et al.* Robust Aqua Material: A Pressure-Resistant Self-Assembled Membrane for Water Purification. *Angewandte Chemie International Edition* **56**, 2203-2207 (2017).

8 Wang, N., Ji, S., Zhang, G., Li, J. & Wang, L. Self-assembly of graphene oxide and polyelectrolyte complex nanohybrid membranes for nanofiltration and pervaporation. *Chemical engineering journal* **213**, 318-329 (2012).

9 Hu, M. & Mi, B. Enabling graphene oxide nanosheets as water separation membranes. *Environmental science & technology* **47**, 3715-3723 (2013).

10 Agenson, K. O., Oh, J.-I. & Urase, T. Retention of a wide variety of organic pollutants by different nanofiltration/reverse osmosis membranes: controlling parameters of process. *Journal of Membrane Science* **225**, 91-103 (2003).

11 Ahmad, A., Ooi, B., Mohammad, A. W. & Choudhury, J. Development of a highly hydrophilic nanofiltration membrane for desalination and water treatment. *Desalination* **168**, 215-221 (2004).

12 Han, Y., Jiang, Y. & Gao, C. High-flux graphene oxide nanofiltration membrane intercalated by carbon nanotubes. *ACS applied materials & interfaces* **7**, 8147-8155 (2015).

13 Han, Y., Xu, Z. & Gao, C. Ultrathin graphene nanofiltration membrane for water purification. *Advanced Functional Materials* **23**, 3693-3700 (2013).

14 Wang, Z. *et al.* Understanding the Aqueous Stability and Filtration Capability of MoS2 Membranes. *Nano letters* **17**, 7289-7298 (2017).
